# Unravelling pathological ageing with brain age gap estimation in Alzheimer’s disease, diabetes and schizophrenia

**DOI:** 10.1093/braincomms/fcaf109

**Published:** 2025-03-11

**Authors:** Maria Fátima Dias, João Valente Duarte, Paulo de Carvalho, Miguel Castelo-Branco

**Affiliations:** CIBIT (Coimbra Institute for Biomedical Imaging and Translational Research), ICNAS, University of Coimbra, 3000-548 Coimbra, Portugal; Institute of Physiology, Faculty of Medicine, University of Coimbra, 3000-548 Coimbra, Portugal; CISUC/LASI – Centre for Informatics and Systems of the University of Coimbra, University of Coimbra, 3030-790 Coimbra, Portugal; CIBIT (Coimbra Institute for Biomedical Imaging and Translational Research), ICNAS, University of Coimbra, 3000-548 Coimbra, Portugal; Institute of Physiology, Faculty of Medicine, University of Coimbra, 3000-548 Coimbra, Portugal; CISUC/LASI – Centre for Informatics and Systems of the University of Coimbra, University of Coimbra, 3030-790 Coimbra, Portugal; Health Research Line, Intelligent Systems Associate Laboratory (LASI), 4800-058 Guimarães, Portugal; CIBIT (Coimbra Institute for Biomedical Imaging and Translational Research), ICNAS, University of Coimbra, 3000-548 Coimbra, Portugal; Institute of Physiology, Faculty of Medicine, University of Coimbra, 3000-548 Coimbra, Portugal; Health Research Line, Intelligent Systems Associate Laboratory (LASI), 4800-058 Guimarães, Portugal

**Keywords:** explainability, deep learning, brain age gap, neuropsychiatric disorders

## Abstract

Brain age gap estimation (BrainAGE), the difference between predicted brain age and chronological age, might be a putative biomarker aiming to detect the transition from healthy to pathological brain ageing. The biomarker primarily models healthy ageing with machine learning models trained with structural magnetic resonance imaging (MRI) data. BrainAGE is expected to translate the deviations in neural ageing trajectory and has been shown to be increased in multiple pathologies, such as Alzheimer’s disease (AD), schizophrenia and Type 2 diabetes (T2D). Thus, accelerated ageing seems to be a general feature of neuropathological processes. However, neurobiological constraints remain to be identified to provide specificity to this biomarker. Explainability might be the key to uncovering age predictions and understanding which brain regions lead to an elevated predicted age on a given pathology compared to healthy controls. This is highly relevant to understanding the similarities and differences in neurodegeneration in AD and T2D, which remains an outstanding biological question. Sensitivity maps explain models by computing the importance of each voxel on the final prediction, thereby contributing to the interpretability of deep learning approaches. This paper assesses whether sensitivity maps yield different results across three conditions related to pathological neural ageing: AD, schizophrenia and T2D. Five deep learning models were considered, each model trained with different MRI data types: minimally processed T_1_-weighted brain scans, and corresponding grey matter, white matter, cerebrospinal fluid tissue segmentation and deformation fields (after spatial normalization). Our results revealed an increased BrainAGE in all pathologies, with a different mean, which is the smallest in schizophrenia; this is in line with the observation that neural loss is secondary in this early-onset condition. Importantly, our findings suggest that the sensitivity, indexing regional weights, for all models varies with age. A set of regions were shown to yield statistical differences across conditions. These sensitivity results suggest that mechanisms of neurodegeneration are quite distinct in AD and T2D. For further validation, the sensitivity and the morphometric maps were compared. The findings outlined a high congruence between the sensitivity and morphometry maps for age and clinical group conditions. Our evidence outlines that the biological explanation of model predictions is vital in adding specificity to the BrainAGE and understanding the pathophysiology of chronic conditions affecting the brain.

## Teaser

Maria *et al*. explore explainability as a strategy to add specificity to brain age gap estimation (BrainAGE) as a putative biomarker. Three clinical conditions are compared: Type 2 diabetes, schizophrenia and Alzheimer’s disease. The results suggest that explainability could be a strategy to decode the disease behind an increased BrainAGE.

## Graphical Abstract

**Graphical Abstract:**
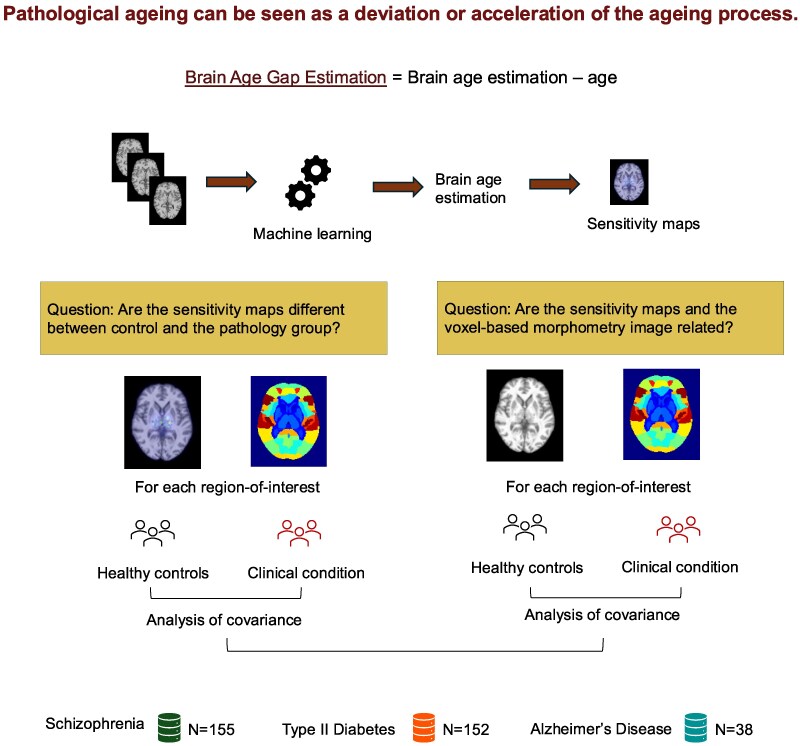

## Introduction

Ageing is a dynamic biological process intrinsically connected to the natural history of multiple diseases.^1,2^ The biological mechanisms behind the transition from healthy to pathological ageing remain to be uncovered in a broad range of conditions. Pathological ageing may share biological pathways with healthy ageing, suggesting that, in some cases, the former might represent an acceleration of the latter. Nevertheless, pathological ageing might also be characterized by disease-specific mechanisms.^3^ The incidence of age-related diseases has been increasing in the last decade. Thus, developing an ageing biomarker that enables healthy ageing and detecting the transition to pathological ageing is paramount. Brain age gap estimation (BrainAGE) is a putative ageing biomarker that aims to identify the onset of pathological ageing of the brain and monitor its progress. BrainAGE corresponds to the difference between predicted brain age and chronological age.^4^ Succinctly, the predicted age is obtained through machine learning algorithms that learn healthy ageing patterns from structural MRI data.^5^ BrainAGE is increased in multiple pathological conditions, for instance, Alzheimer’s disease, mild cognitive impairment, mood disorders, epilepsy and schizophrenia, among others,^5,6^ suggesting that its sensitivity is high, although these prior approaches suffer from a lack of specificity. The guidelines of the American Federation for Aging Research state that an ageing biomarker should be able to detect and specify the pathology in its early stages.^7^ Thus, adding specificity to the prediction is essential to validate the BrainAGE as an ageing valuable biomarker in clinical practice. Currently, state-of-the-art models on brain age belong to the category of deep learning, which are considered black-box models.^6^ Understanding the contribution of different brain regions on a given prediction is essential to accepting deep learning models in clinical practice, and it might be the key to adding specificity to this putative biomarker—BrainAGE. Two strategies have been used to address this issue: to predict age locally and to compute the sensitivity maps. Local brain age models predict the age per patch or region rather than using the entire volume.^8-11^ Thus, this strategy assigns an age per region, which enables the identification of accelerated ageing areas. However, the performance of such models is poor compared to global brain age models, which might be caused by insufficient information to derive accurate predictions. Another approach towards explainability is entailed by sensitivity or saliency maps.^12-14^ Sensitivity maps unveil the influence that each voxel may exert on a prediction. These maps have been explored in the brain age context, and the results are congruent: the regions with a higher contribution to the predictions are located around the ventricles.^12-14^ Nevertheless, to the best of our knowledge, no study has assessed these maps to discern the differentially elevated regional BrainAGE values in different pathologies. In this work, we aim to uncover the brain age models’ predictions and verify whether the origin of an elevated BrainAGE is different across three chronic pathologies: Alzheimer’s disease, schizophrenia, and Type 2 diabetes (T2D). These diseases have different aetiologies and are associated to specific periods of lifetime, Alzheimer’s disease manifests in late adulthood, T2D in mid to later adulthood and schizophrenia in early adulthood or late adolescence. Furthermore, two morphological hallmarks are reflected in all three pathologies: higher brain atrophy and ventricle enlargement. Notably, BrainAGE has been shown to reflect the atypical healthy trajectory ageing of the brain in these conditions, in distinct previous studies,^15-20^ while biological specificity remained to be uncovered.

Alzheimer’s disease is the most prevalent neurodegenerative disease, and ageing is a major risk factor for developing the disease. The structural brain changes in Alzheimer’s disease involve a pronounced loss of grey matter (GM) and an exacerbated increase in CSF, particularly noticeable in the ventricles.^21,22^ T2D is a metabolic disorder characterized by decreased insulin production and/or increased insulin resistance that impacts glucose level regulation. Uncontrolled glucose levels have been associated with abnormal loss of GM, an increase in the CSF and changes in the gyrification patterns compared to healthy controls.^23-25^ Both Alzheimer’s disease and T2D have been associated with the controversial concept of brain insulin resistance, which may result in regional hypometabolism. While the pathogenesis of Alzheimer’s disease remains to be uncovered, some researchers suggest that insulin resistance prompts the accumulation of both beta-amyloid and tau, therefore suggesting that Alzheimer’s disease is ‘the diabetes of the brain’ or Type 3 diabetes.^26^ Nonetheless, it should be highlighted this account is still highly disputed. Lastly, schizophrenia is a late neurodevelopmental mental disorder characterized by psychotic manifestations. This disease is characterized by atypical neural connectivity both at anatomical and functional levels, as well as an enlargement of the subcortical structures alongside a reduction in cortical volume and thickness.^27-30^

In brief, this work aims to:

1. Compare the BrainAGE in different pathologies across different input image modalities as a tool to understand the respective underlying neurobiology;
2. Assess sensitivity maps across different pathologies to test the hypothesis that biological explainability is distinct—this is critical for the hypothesized relation between T2D and Alzheimer’s disease; and
3. Compare the results obtained with the computation of sensitivity maps with those obtained with the morphological maps as a validation approach.

## Materials and methods

### Data

**Figure 1:**
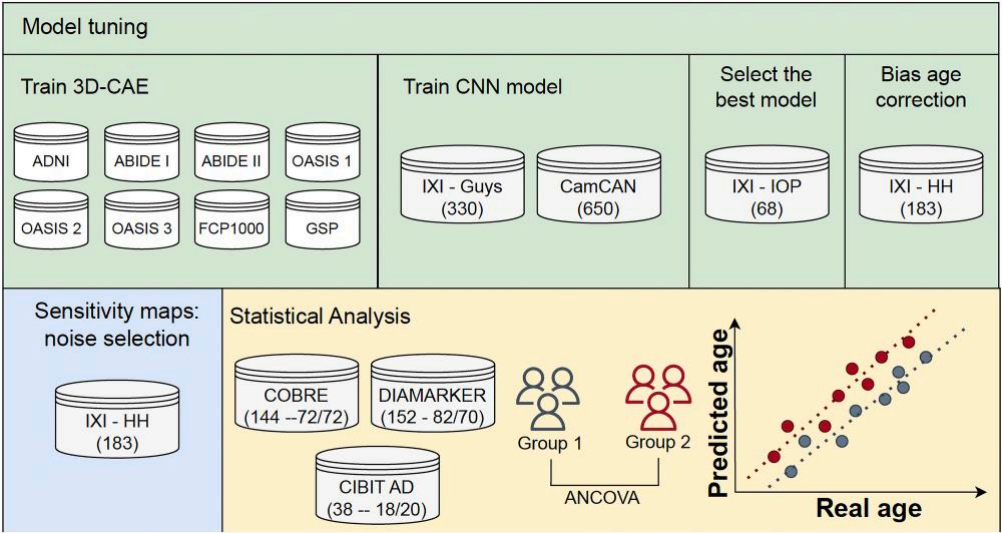
**Scheme of the methodology used in this work**. Firstly, a 3D Convolutional Autoencoder (3D-CAE) was trained per modality with multiple open-source datasets: Autism Brain Imaging Data Exchange (ABIDE), ABIDE II, Brain Genomics Superstruct (GSP), Open Access Series of Imaging Studies (OASIS)-1, OASIS-2, OASIS-3, 1000 Functional Connectomes Project (FCP1000) and Alzheimer’s Disease Neuroimaging Initiative (ADNI). Then, the encoder weights of 3D-CAE were transferred to the corresponding convolutional neural network (CNN) regression model, which was, in turn, trained with the data from the Cambridge Centre for Ageing and Neuroscience (CamCAN) repository and from Guys of the Information eXtraction from Images (IXI) dataset. The predictions were corrected using the Hammersmith Hospital (HH) dataset. The sensitivity maps were generated using the uncorrected predictions. Finally, the sensitivity maps and the BrainAGE were compared for each clinical dataset (schizophrenia—Center for Biomedical Research Excellence (COBRE) dataset; diabetes type II—DIAMARKER dataset; and Alzheimer’s disease—Coimbra Institute for Biomedical Imaging and Translational Research (CIBIT) AD) between the clinical condition and the healthy controls.

This study considered 13 datasets: 11 open-source data repositories and two local datasets. Table 1 provides a summary of demographic information for each of the datasets used in this study, except for training of the 3D Convolutional Autoencoder (3D-CAE), for this case, the demographic information is provided in Supplementary Table 1. An overview scheme of the methodology is shown in Fig. 1, the first step of this methodology is the training of the 3D-CAE. To perform this step, eight of the datasets [Autism Brain Imaging Data Exchange (ABIDE) I,^31^ ABIDE II,^32^ Brain Genomics Superstruct (GSP),^33^ Open Access Series of Imaging Studies (OASIS)-1,^34^ OASIS-2,^35^ OASIS-3,^36^ 1000 Functional Connectomes Project (FCP1000)^37^ and Alzheimer’s Disease Neuroimaging Initiative (ADNI)^38^] were considered, which comprised 29 478 images from 75 sites. Two open-source repositories were considered in the model tuning phase: Cambridge Centre for Ageing and Neuroscience (CamCAN)^39,40^ and the Information eXtraction from Images (IXI).^41^ IXI are multi-site repositories with data collected independently from three hospitals in London: Guys Hospital, Hammersmith Hospital (HH) and IOP (Institute of Psychiatry). Finally, to evaluate the performance of the models on the three diseases of interest in this study, three distinct datasets were employed, one dataset per pathology. For schizophrenia, the open-source repository Center for Biomedical Research Excellence (COBRE) dataset was used, whereas local datasets for T2D^42^ and Alzheimer’s disease^43^ were considered. Additionally, for the Alzheimer’s disease analysis, a parallel analysis was performed using the OASIS4 dataset.^44^ The results are detailed in Supplementary Section 2.3, including Supplementary Figs 3–10 and Supplementary Tables 12–15.

**Table 1:** Demographics of the datasets considered for model tuning (CamCAN and IXI) and to assess BrainAGE across different clinical conditions (schizophrenia, T2D and Alzheimer’s disease)

Dataset	Total	Number of males	Mean and standard deviation [years]	Min age [years]	Max age [years]	Number of controls
CamCAN	642	318	54.23 ± 18.6	18	88	642
IXI—Guys	312	139	50.73 ± 15.98	20.07	86.20	312
IXI—HH	179	85	47.63 ± 16.61	20.17	81.94	179
IXI—IOP	67	24	42.13 ± 16.60	19.98	86.32	67
Schizophrenia	144	107	37.11 ± 12.82	18	65	72
T2D	152	73	54.68 ± 9.61	40	76	82
Alzheimer’s disease	38	19	66.08 ± 6.66	52	76	18

The data from the T2D and Alzheimer’s disease were collected under the scope of two studies approved by the Ethics Committee of the Faculty of Medicine, University of Coimbra. All subjects participated voluntarily and gave their informed written consent for the study, following the tenets of the Declaration of Helsinki. In both studies, subjects were clarified about the nature and possible implications of the study.

### Pre-processing

MRI structural T_1_-weighted scans were considered in this study. The images were pre-processed using the *CAT12* default pre-processing pipeline (‘Segment’) and SPM12, due to the low pre-processing time and high reliability they provide, in MATLAB environment. The CAT12, SPM12^45^ and MATLAB versions used were version 1742, v7771 and R2020a (9.8.0.1323502), respectively. The CAT12 framework requires images to be aligned in the anterior and posterior commissures planes, which was performed using the ATRA toolbox (v2.0). Furthermore, CAT12 pre-processing guidelines recommend that, for images of individuals aged 18 years or less, a personalized template should be created for the registration and segmentation steps. Therefore, these templates were created using the Cerebromatic toolbox^46^ and TOM8 toolbox,^47^ respectively. The default CAT12 template was considered for images of adult participants, whereas children’s images were registered to a personalized template. The images considered in this work were registered in the MNI space, with the dimensions 121 × 145 × 121. Five types of information extracted from the MRI structural images were considered per subject, i.e. the minimally processed images; the T_1_-weighted segmented in GM, white matter (WM) and CSF; and the deformation fields resulting from the normalization (to MNI space) procedure. These different information types, extracted from the same MRI images, will be designated throughout this paper as modalities. Minimally processed T_1_-weighted images attain the best performance compared to using a single tissue type or a combination of tissues.^48,49^ This is expected since these images contain all brain structures rather than just a portion of the information. Nevertheless, each disease affects each tissue differently. Alzheimer’s disease primarily affects GM, and the loss of this tissue results in an exaggerated enlargement of the ventricles.^21,22^ Schizophrenia also affects GM (and possibly WM).^50,51^ Concerning T2D, this condition mainly affects the vascular system, which impacts WM earlier than GM, regarding small vessel disease.^24,25^ Moreover, an increase in CSF was also reported in diabetes compared to healthy controls. Therefore, we decided to investigate whether using tissue-level images would provide more insights than minimally processed images. Regarding deformation fields, this feature yielded promising results compared to WM and CSF.^52^ Therefore, we decided to investigate whether this source of information would yield complementary insights to traditional brain tissues.

### Brain age model tuning

Brain age models were developed leveraging off-the-shelf transfer learning from 3D-CAE, which has been suggested to yield superior results compared to training a convolutional neural network (CNN) from scratch.^53^ Therefore, the creation of each brain age model encompasses the training of two models, the 3D-CAE and then the brain age CNN. The architectures considered for the 3D-CAE and regression CNN were the ones considered in a previous study.^53^ Regarding the 3D-CAE, as described above, all data from the ABIDE I, ABIDE II, GSP, OASIS-1, OASIS-2, OASIS-3, FCP1000 and ADNI repositories were considered to train and validate the models. All images from these datasets were included, regardless of the condition of the participants, which comprised a total of 29 478 images, out of which 250 instances were used to validate and select the best model. The 3D-CAE models were trained for over 50 epochs with a batch size of 16, except for the deformation field modality. For the deformation fields, the loss of the 3D-CAE model diverged after 20 epochs, requiring an increase in batch size to 56 and an extension of the training to 150 epochs. The autoencoders were optimized using the Mean Squared Error as the loss. The 3D-CAE model selected was the one that exhibited the highest performance on the validation set during the last 30 training epochs.

The 3D-CAE encoder weights were reused on the corresponding brain age CNN model. The transferred weights were frozen, and only the layers in which the weights were randomly initialized were updated during training, as described by Dias *et al.*^53^ The CamCAN and the IXI repository were considered to train, select and evaluate the brain age CNN models. The brain age CNN models were trained with 954 images from the CamCAN repository and using Guy’s data from the IXI repository. All the brain age models were trained over 150 epochs, and the data from the IOP of the IXI dataset were used to select the best model of the last 30 training epochs. Finally, to correct the model bias of age models, the data from the HH site were used to adjust the bias using the approach proposed by Beheshti *et al*.^54^

### Statistical analysis

#### BrainAGE on different pathologies

The BrainAGE was investigated per pathology dataset (schizophrenia, T2D and Alzheimer’s disease) and modality. For each case, the BrainAGE was compared between clinical conditions (disease status versus healthy controls) using an analysis of covariance (ANCOVA) controlled for age and gender. Age should be accounted for in the analysis since brain age models often report an age bias, and models tend to underestimate and overestimate the age of young and older subjects, respectively.^54^ Despite bias correction being performed, the bias might not be completely removed and, therefore, the age was still accounted for in statistical comparisons. An ANCOVA was performed with and without bias correction.

#### Sensitivity maps generation

Sensitivity maps were computed using the SmoothGrad approach.^55^ Sensitivity maps tend to be noisy, thus to overcome this issue, SmoothGrad applies Gaussian noise to the input and computes the corresponding sensitivity maps. The procedure is performed multiple times, and the final sensitivity map is the average of the multiple noisy sensitivity maps. The level of noise that should be added depends on the input type. In this work, five inputs were considered, i.e. minimally processed, GM, WM, CSF and deformation fields. Thus, the appropriate noise level should be selected for each one. To select the best noise structure, an assumption was made to ensure the one that maximized the correlation between the age and sensitivity maps. To perform this analysis, the data from the HH of the IXI dataset were considered. The sensitivity maps were computed for each subject and parcelled according to the neuromorphometrics atlas. For each region of interest (ROI), the mean value was computed. Then, for each ROI, the Pearson correlation was computed between age and the ROI sensitivity. Different noise levels were evaluated for the five input types, with values ranging from 0% to 50%, with a step of 2%.

#### Sensitivity maps and morphometry on different pathologies

The morphometric and sensitivity maps were assessed individually (stage 1) and compared with each other (stage 2). Both analyses were performed at an ROI level. Thus, the modality images and sensitivity maps were parcelled according to the neuromorphometrics template and for each ROI the mean was computed.

Stage 1 aims to understand the relation of the ROI value with age and health condition. In the morphometric case, the analysis portrays the regions that yield significant volume changes with age and clinical conditions. To achieve this, an ANCOVA was performed with the ROI value (sensitivity or morphometric measure) as the dependent variable, the clinical condition as the group and age as a covariate. The *P*-values were corrected for multiple comparisons using the false discovery rate.^56^ This analysis enables the assessment of the regions in which the sensitivity and/or or the morphometry correlates with age and which regions were sensitive to clinical conditions.

Stage 2 aims to assess whether the results from the morphometric and sensitivity maps were congruent. To achieve this, the Jaccard coefficient was considered. Jaccard index is the ratio between the number of significant regions in the analyses and the total number of significant regions in both analyses, thus measuring the overlap between the two maps.

## Results

### Brain age models performance

The results regarding mean absolute error (MAE) for the IOP and HH data and the correlation between MAE and BrainAGE with and without bias correction are displayed in Supplementary Table 2. The model selection process was based on the IOP dataset, and the MAE on minimally processed, GM, WM, CSF and deformation fields was 4.66, 5.36, 6.10, 5.61 and 5.48 years, respectively. Subsequently, the model was evaluated on an external dataset, the data from the HH of the IXI dataset. The results, on HH data, yield a lower MAE of 4.46, 5.75, 5.26, 5.44 and 6.53 years for minimally processed, GM, WM, CSF and deformation fields, respectively.

Concerning the age bias, the findings reveal as reported in the literature^54^ that, without any bias correction, a significant correlation exists between BrainAGE and age for all image types in both sites. Regarding the corrected age predictions, the bias is corrected in all modalities on the HH set, this finding is expected since this dataset was the one used for the correlation. In the case of the IOP data, the correlation decreased in all cases and is completely corrected for three modalities (minimally processed, CSF and deformation fields).

#### Schizophrenia

**Figure 2:**
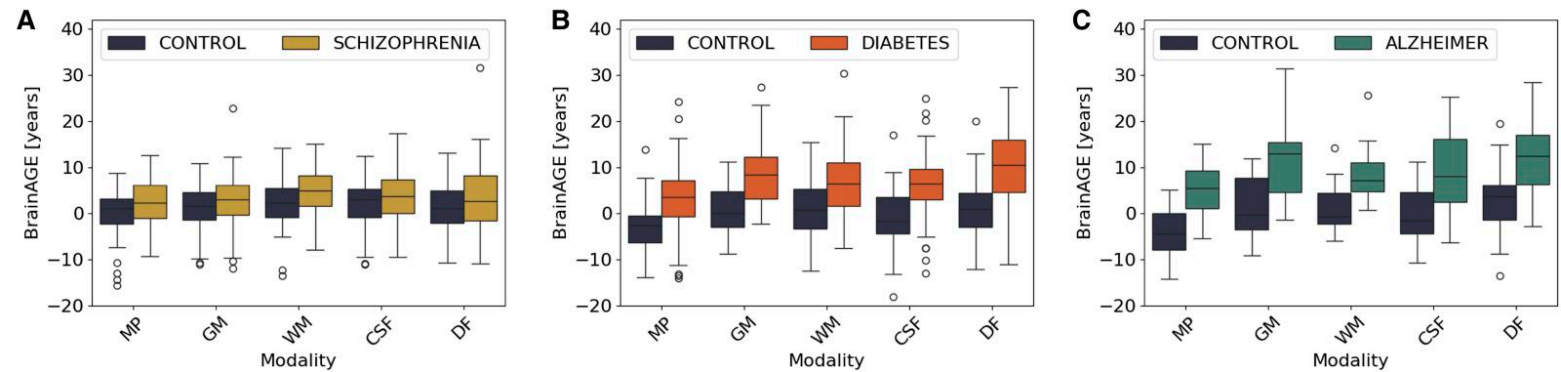
**Brain age gap estimation (BrainAGE) difference boxplot**. Boxplot that represents the quantiles of the difference between age prediction and chronological age for the different pathologies considered in this work: (**A**) schizophrenia, (**B**) Type 2 diabetes (T2D) and (**C**) Alzheimer’s disease. For the different brain age models: minimally processed (MP), grey matter (GM), white matter (WM), CSF and deformation fields (DF).

A summary of the MAE and BrainAGE for different modalities can be found in Fig. 2 and in Supplementary Table 3. The BrainAGE difference across groups is 2.40, 1.47, 2.82, 1.92 and 2.37 years on minimally processed image, GM, WM, CSF and deformation fields, respectively. Thus, in this chronic mental disorder, the BrainAGE for the schizophrenia group is higher than in the control group. The ANCOVA results for the BrainAGE are in Table 2. The results indicate the significant BrainAGE on all modalities except GM. Furthermore, the bias on BrainAGE value was significant on minimally processed, GM and WM.

**Table 2:** ANCOVA results comparing the BrainAGE and controlling for age

	Tissue	Source	SS	F	*P*-value	np2
Schizophrenia	MP	Condition	259.37	10.87	**0**.**0012**	0.072
		Age	8.6	0.36	0.55	0.0026
		Gender	351.05	14.71	**0**.**00019**	0.095
	GM	Condition	111.47	3.58	0.06	0.025
		Age	30.56	0.98	0.32	0.007
		Gender	173.02	5.56	**0**.**02**	0.038
	WM	Condition	280.88	10.77	**0**.**0013**	0.071
		Age	3.27	0.13	0.72	0.00089
		Gender	20.13	0.77	0.38	0.0055
	CSF	Condition	172.75	5.88	**0**.**017**	0.04
		Age	3.66	0.12	0.72	0.00089
		Gender	338.26	11.51	**0**.**0009**	0.076
	DF	Condition	194.39	4.6	**0**.**034**	0.032
		Age	6.99	0.17	0.68	0.0012
		Gender	12.95	0.31	0.58	0.0022
	MP	Condition	1600.77	41.72	**1.43 × 10^−9^**	0.22
		Age	1.3	0.034	0.85	0.00023
		Gender	64.4	1.68	0.2	0.011
	GM	Condition	1549.64	46.09	**2.55 × 10^−10^**	0.24
		Age	0.18	0.0052	0.94	**3.52 × 10^−5^**
		Gender	8.34	0.25	0.62	0.0017
T2D	WM	clinical Condition	979.76	22.52	**4.87 × 10^−6^**	0.13
		Age	35.74	0.82	0.37	0.0055
		Gender	18.98	0.44	0.51	0.0029
	CSF	Condition	1665.07	40.23	**2.59 × 10^−9^**	0.21
		Age	22.61	0.55	0.46	0.0037
		Gender	36.17	0.87	0.35	0.0059
	DF	Condition	1414.07	27.46	**2.59 × 10^−7^**	0.16
		Age	77.55	1.51	0.22	0.01
		Gender	173.6	3.37	0.068	0.022
Alzheimer’s disease	MP	Condition	777.4	21.42	**5.19 × 10^−5^**	0.39
		Age	0.051	0.0014	0.97	**4.11 × 10^−5^**
			4.37	0.12	0.73	0.0035
	GM	Condition	935.58	15.03	**0**.**00046**	0.31
		Age	0.088	0.0014	0.97	**4.15 × 10^−5^**
			3.09	0.05	0.82	0.0015
	WM	Condition	519.28	15.55	**0**.**00038**	0.31
		Age	5.31	0.16	0.69	0.0047
		Gender	3.52	0.11	0.75	0.0031
	CSF	Condition	887.17	13.23	**0**.**0009**	0.28
		Age	7.02	0.1	0.75	0.0031
		Gender	0.44	0.0065	0.94	0.00019
	DF	Condition	751.17	11.32	**0**.**0019**	0.25
		Age	3.24	0.049	0.83	0.0014
		Gender	208.11	3.14	0.086	0.084

An ANCOVA was performed per disease and modality (minimally processed image, GM, WM, CSF and deformation fields) of healthy controls versus subjects diagnosed with schizophrenia, T2D and Alzheimer’s disease. The significant values are represented in bold.

#### Type 2 diabetes

The age prediction results for the T2D dataset are depicted in Fig. 2. A summary of the results achieved for the mean MAE and BrainAGE for the health group versus T2D is in Supplementary Table 4. We found that the predicted age in the T2D group is higher than healthy controls. The mean BrainAGE difference between groups is 6.75, 7.76, 5.59, 7.2 and 8.47 years for minimally processed image, GM, WM, CSF and deformation fields, respectively. The statistical analysis for the BrainAGE is in Table 2. The results outline that the statistical difference is significant for all modalities. Concerning the age bias, the ANCOVA results exhibited no age bias on BrainAGE.

#### Alzheimer’s disease

Figure 2 shows the relation between true age and corrected age predictions across various modalities within the Alzheimer’s disease dataset. Supplementary Table 5 in Section 2.2 details the mean MAE and BrainAGE for the corrected predictions. The results reveal that, on average, the BrainAGE is higher in Alzheimer’s disease patients. Specifically, the mean BrainAGE difference between Alzheimer’s disease patients and healthy controls is 9.04, 9.96, 7.43, 9.69 and 9.04 years for minimally processed, GM, WM, CSF and deformation fields, respectively. Thus, for all modalities except WM, the BrainAGE in Alzheimer’s disease patients is, on average, 9+ years higher compared to healthy controls. The ANCOVA results in Table 2 confirm the significant differences in BrainAGE between the two groups on all modalities. Regarding age bias, the ANCOVA analysis of the BrainAGE suggests no bias in this dataset.

### Age

#### Relationship of ROI-based morphometry with age

**Figure 3:**
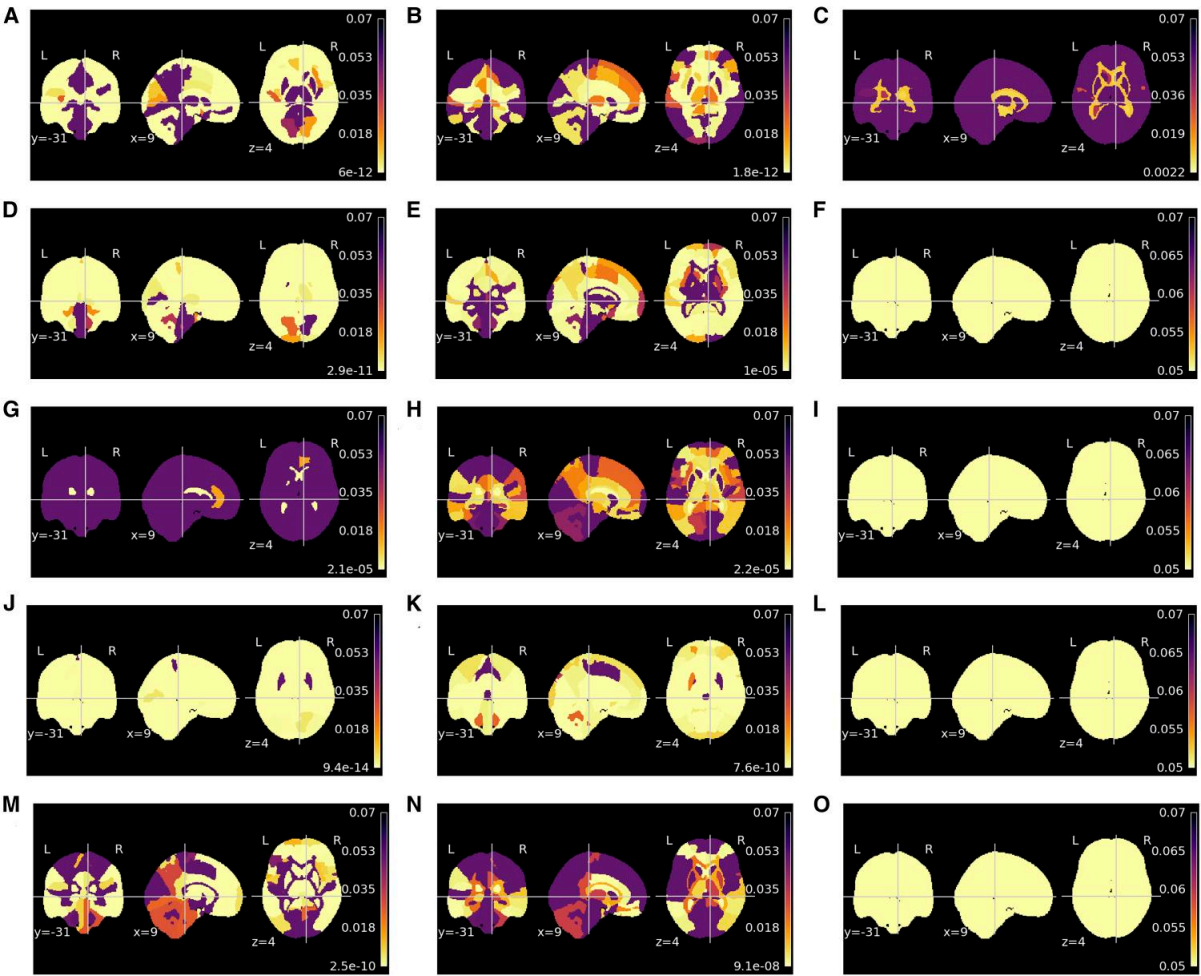
**Region-of-interest (ROI) analysis of covariance (ANCOVA) *P*-value results for age on morphometric maps**. The ANCOVA compared the morphometric map ROI mean of clinical conditions [health controls/pathology] and controlling for age. The pathologies assessed were schizophrenia (**A**, **D**, **G**, **J** and **M**) [72 controls/72 schizophrenia], Type 2 diabetes (T2D) (**B**, **E**, **H**, **K** and **N**) [82 controls/70 T2D] and Alzheimer’s disease (**C**, **F**, **I**, **L** and **O**) [18 controls/20 Alzheimer’s disease]. Different morphometric maps assessed were minimally processed (**A–C**), grey matter (**D–F**), white matter (**G–I**), cerebrospinal fluid (**J–L**) and deformation fields (**M–O**).

The regions showing significant regression coefficients for the age factor, or, in other words, regions whose value correlates with age in a significant manner are depicted in Fig. 3. The mean morphometric maps for each dataset are shown in Supplementary Fig. 1. The proportion of significant regions in each modality and dataset is in Supplementary Table 6 in Section 2.2. For schizophrenia and T2D, the results indicate that almost all regions are significant for the GM and CSF modalities. On minimally processed modality, between one-third and half of the regions are considered significant, whereas on the deformation fields, around half of the regions yield a significant correlation with age. The WM yield higher morphometry differences across the schizophrenia and T2D cases, while on T2D, only the ventricles are considered to yield volume differences with age, and on the schizophrenia cases, more than half of the regions are considered significant.

The Alzheimer’s disease dataset yields the lowest number of regions significantly correlated with age. The modality with the highest number of significant regions is minimally processed images with 8.57% being significant regions. In all other modalities, no region is considered to be significant. These results might be explained by the narrow age range of the dataset as well as the number of instances in the dataset. The dataset contains only 38 subjects aged between 52 and 76 years. Thus, the statistical power of the data to detect variations might be smaller. Finally, it may also be possible that Alzheimer’s disease pathology overrides age-related changes.

#### Sensitivity maps on age prediction

The analysis of the relation between sensitivity maps and noise is discussed in Supplementary Section 2.4. The results reveal that each modality yields the maximum correlation at a different noise level. Thus, the noise level considered was different across modalities and corresponded to the noise yielding the highest correlation value between sensitivity maps and age on the HH data.

**Figure 4:**
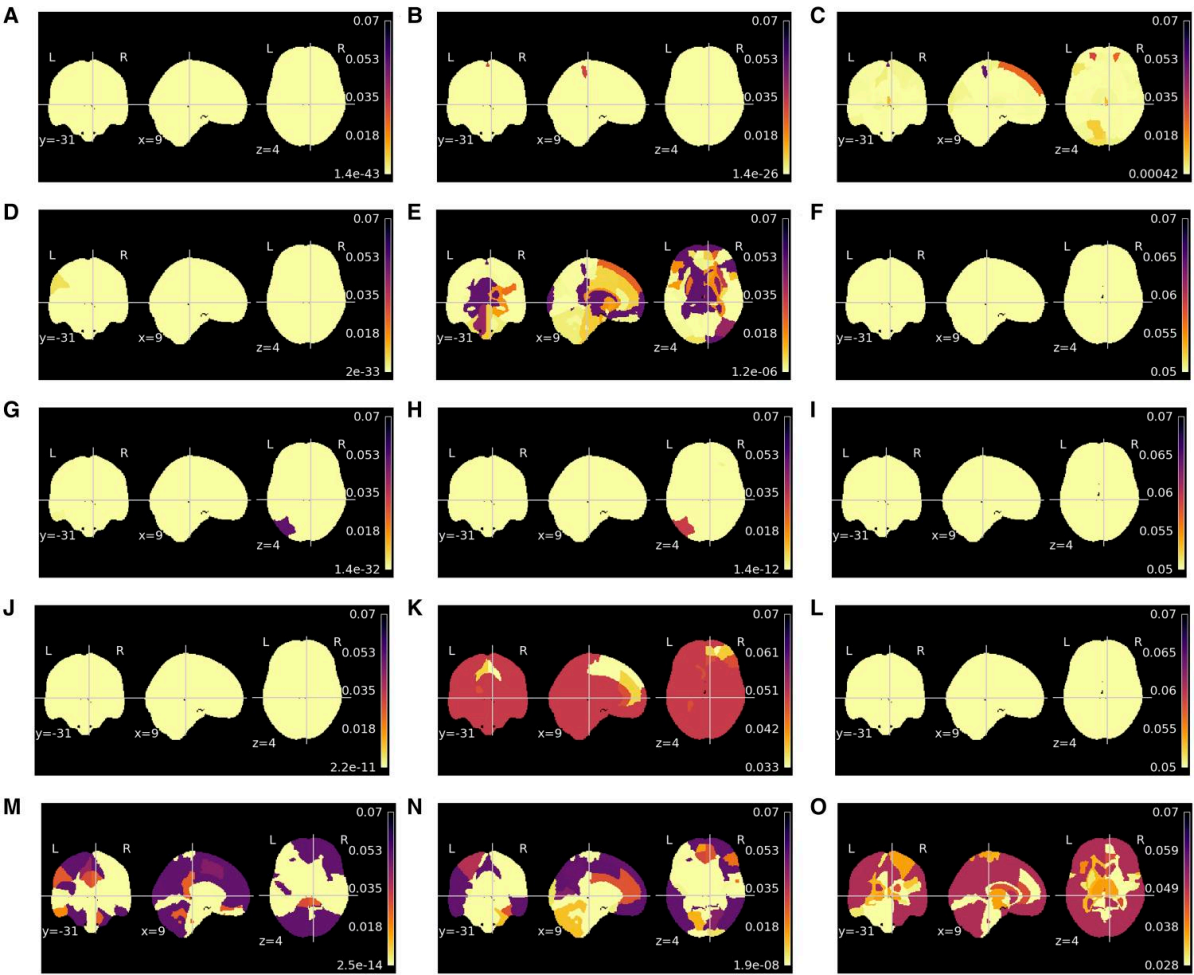
**Region-of-interest (ROI) analysis of covariance (ANCOVA) *P*-value results for age on sensitivity maps**. The ANCOVA compared the morphometric map ROI mean of clinical conditions (health controls versus pathology) and controlling for age. The pathologies assessed were schizophrenia (**A**, **D**, **G**, **J** and **M**) [72 controls/72 schizophrenia], Type 2 diabetes (T2D) (**B**, **E**, **H**, **K** and **N**) [82 controls/70 T2D] and Alzheimer’s disease (**C**, **F**, **I**, **L** and **O**) [18 controls/20 Alzheimer’s disease]. Different sensitivity maps derived from brain age models trained with minimally processed (**A–C**), grey matter (**D–F**), white matter (**G–I**), cerebrospinal fluid (**J–L**) and deformation fields (**M–O**) were assessed.

The mean sensitivity maps for each dataset are presented in Supplementary Fig. 2. The results indicate that regions with higher sensitivity are around the ventricles in all modalities, possibly due to their fast change in shape during ageing. This finding is congruent with other published works.^12-14^ The significant regions concerning the sensitivity for the age factor are shown in Fig. 4, and the percentage of the significant regions is in Supplementary Table 7. The results suggest that the sensitivity of a region correlates with age on all regions in the minimally processed image modality, in all datasets except the Alzheimer’s disease dataset, in which 99.29% of the regions were considered significant. In the case of GM and CSF modalities, all regions were significant for the schizophrenia dataset. On the T2D, the correlation of sensitivity with age was statistically significant in 72.14% and 10.00% of the regions for GM and CSF modalities, respectively. Similarly, to the morphometric results, on the Alzheimer’s disease, no region is correlated with age on GM, WM and CSF. However, in this case, almost all and 30% of regions are considered significant on minimally processed and deformation fields, respectively.

#### Sensitivity versus morphometry maps on age prediction

Some ROIs yield a significant age correlation both on morphometry and sensitivity maps analysis, Supplementary Table 8 shows the Jaccard index between significant regions in both analyses. The findings reveal that the overlap depends upon the dataset and modality. An almost perfect agreement in both analyses is reported on the GM and CSF modalities on the schizophrenia. GM is also the modality with the highest overlap, a Jaccard index of 0.72, on the T2D. Concerning the minimally processed image, the Jaccard of 0.75 and 0.58 is reported for the schizophrenia and T2D cases, respectively. WM has a good agreement comparing the morphometry and sensitivity map results on T2D but not on schizophrenia. Around one-third of the regions are significant in both analyses regarding the deformation fields. Lastly, consistent with the preceding results, the Alzheimer’s disease dataset yields a reduced overlap between the regions. Nevertheless, it should be highlighted that the agreement is perfect on CSF, WM and GM, and no region is considered significant in either analysis.

### Clinical group comparisons

#### Morphometry

**Figure 5:**
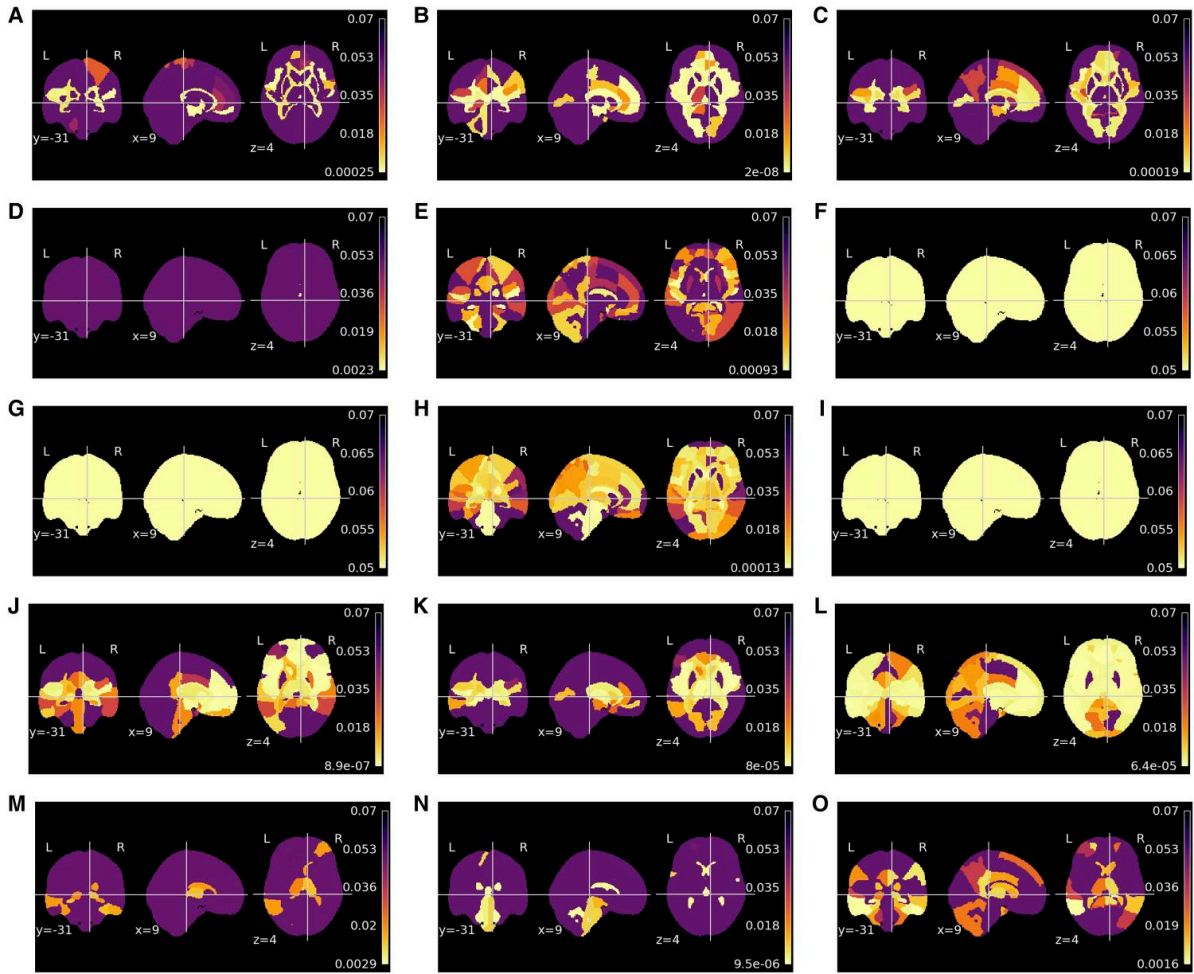
**Region-of-interest (ROI) analysis of covariance (ANCOVA) *P*-value results for clinical condition on morphometric maps**. The ANCOVA compared the morphometric map ROI mean of clinical conditions (health controls versus pathology) and controlling for age. The pathologies assessed were schizophrenia (**A**, **D**, **G**, **J** and **M**) [72 controls/72 schizophrenia], Type 2 diabetes (T2D) (**B**, **E**, **H**, **K** and **N**) [82 controls/70 T2D] and Alzheimer’s disease (**C**, **F**, **I**, **L** and **O**) [18 controls/20 Alzheimer’s disease]. Different morphometric maps assessed were minimally processed (**A–C**), grey matter (**D–F**), white matter (**G–I**), cerebrospinal fluid (**J–L**) and deformation fields (**M–O**).

The statistically significant ROIs across groups in the ANCOVA are depicted in Fig. 5, the percentage of significant regions is detailed in Supplementary Table 9. Compared with the equivalent age results, in general, fewer regions are considered significant for the health condition. The exceptions include the WM on the T2D that yielded more than 10% of significant ROIs for the clinical group condition than for the age. On the Alzheimer’s disease dataset, concerning the age factor, no region is significant on CSF and deformation fields and on minimally processed only 8.57%, whereas on the clinical group condition factor, 30%, 85% and 30% are considered significant regions, respectively.

#### Sensitivity maps

**Figure 6:**
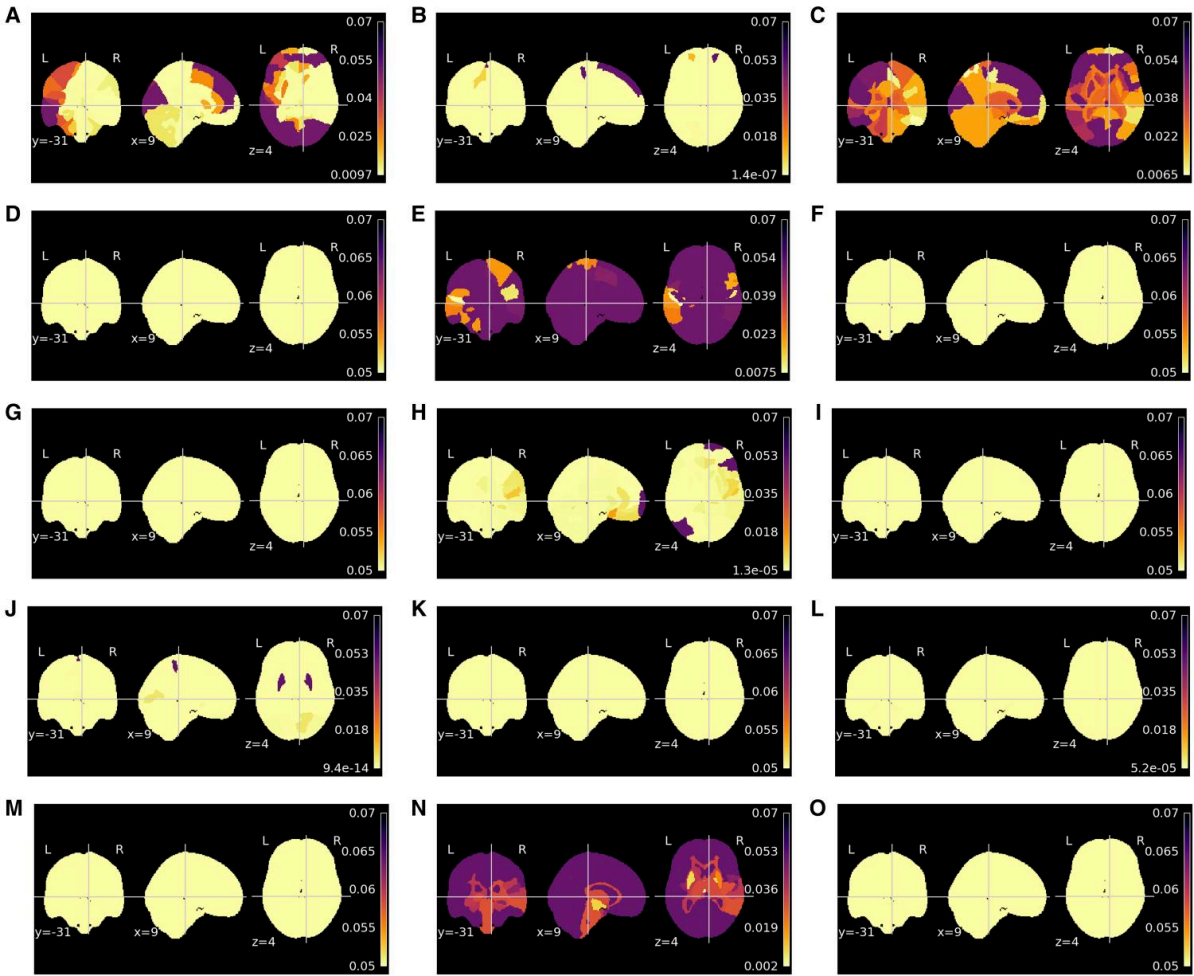
**Region-of-interest (ROI) analysis of covariance (ANCOVA) *P*-value results for clinical condition on sensitivity maps**. The ANCOVA compared the sensitivity map ROI mean of clinical conditions (health controls versus pathology) and controlling for age. The pathologies assessed were schizophrenia (**A**, **D**, **G**, **J** and **M**) [72 controls/72 schizophrenia], Type 2 diabetes (T2D) (**B**, **E**, **H**, **K** and **N**) [82 controls/70 T2D] and Alzheimer’s disease (**C**, **F**, **I**, **L** and **O**) [18 controls/20 Alzheimer’s disease]. Different sensitivity maps derived from brain age models trained with minimally processed (**A–C**), grey matter (**D–F**), white matter (**G–I**), cerebrospinal fluid (**J–L**) and deformation fields (**M–O**) were assessed.

Regions whose sensitivity varies with the condition are depicted in Fig. 6 for each dataset, whereas Supplementary Table 10 summarizes the percentage of significant regions. The analysis suggests that clinical conditions significantly influence sensitivity maps. Minimally processed consistently has a high number of significant regions on all datasets; the percentage of significant regions is 80%, 97.86% and 69.29% for schizophrenia, T2D and Alzheimer’s disease, respectively. Consequently, the overlap between the significant regions on this modality is also high. Concerning the other modalities, the results across pathologies evidence much larger dissimilarities. GM modality only yields significant differences comparing healthy controls with T2D; in this case, 18.57% of regions are considered significant, encompassing the cortical ribbon. Regarding WM, almost all regions (94.29%) demonstrate sensitivity differences between healthy controls and the T2D group. In contrast, no WM region is considered significant when comparing schizophrenia or Alzheimer’s disease with healthy controls. The CSF results suggest that all regions have a significant role in predicting a higher age when comparing the results of the Alzheimer’s disease group with healthy controls. Nevertheless, comparing the sensitivity of T2D or schizophrenia with healthy controls, no significant regions are significant for CSF. Lastly, the deformation field modality yields significant differences only in sensitivity comparing T2D with healthy controls.

#### Morphometric results versus sensitivity maps on pathologies

The overlap between morphometric and sensitivity maps analyses is shown in Supplementary Table 11. In general, the findings outline a low agreement between the morphometry and sensitivity maps, with some exceptions. The two highest Jaccard scores are the CSF and WM on the Alzheimer’s disease and T2D, respectively. Notably, both analyses have a high number of significant regions. The minimally processed images yield a similar overlap score across all datasets, around one-third of the significant ROIs on both analyses. Moreover, although the Jaccard index is zero on the Alzheimer’s disease dataset on GM and WM and the WM on the schizophrenia dataset, in both analyses, no region is considered significant; consequently, the match is perfect. Similarly, on the GM on the schizophrenia dataset, only 1.43% of the regions are considered significant, while on the sensitivity maps, no region yielded significant results; thus, in this case, the match is almost perfect.

## Discussion

The main finding of this article is that the explanation of brain age predictions, based on sensitivity maps, allows the identification of regional specificity of BrainAGE across pathologies. Furthermore, sensitivity maps provide a pathophysiological differentiation between Alzheimer’s disease and T2D. BrainAGE is significant for all the three pathologies considered (Alzheimer’s disease, schizophrenia and T2D) compared to healthy controls, yet the mean BrainAGE is different across pathologies. Alzheimer’s disease yields the highest mean BrainAGE (around 9 years), followed by T2D (around 5 years) and finally by schizophrenia (around 2 years). This result might be explained by the degree of structural changes in each one of the pathologies. Although no prior existing studies compare the structural changes of the three diseases, our data are consistent with the notion that schizophrenia has less direct neural loss compared to T2D, and T2D has, in turn, less structural changes and distinct regional pathology as compared to Alzheimer’s disease. Alzheimer’s disease and T2D are characterized by brain neurodegenerationn,^21,24,25^ which is not the case for schizophrenia.^57,58^ This hypothesis is corroborated by the morphometry analysis conducted in this study, the results outline that T2D yields, on average, more significant ROIs than schizophrenia when comparing the ROIs of the segmented images. Therefore, the BrainAGE might reflect the degree of pathological ageing of the brain.

The morphometric and sensitivity maps yield congruent results on the regions that are age-sensitive. The morphometric results outline that for all modalities (except the WM on the schizophrenia) more than 50% of the regions have significant changes with age on the T2D and schizophrenia. The sensitivity maps observe the same tendency (except for the CSF on the T2D). The overlap between the explainability and morphometry significant regions for age is almost perfect in some cases, for instance, on GM and CSF modalities of schizophrenia, and in other cases, it is around half or more of the overlap between the regions. Furthermore, on the Alzheimer’s disease dataset, no region is considered significant on morphometric and sensitivity maps on the GM, WM and CSF, which is also congruent.

Morphometry results endorse sensitivity maps concerning the regions with different importance across health conditions. On WM tissue, almost all the regions seem to exert a different importance on the prediction comparing the T2D group with the healthy group, a similar finding is outlined with the CSF when analysing the sensitivity differences between Alzheimer’s disease and healthy controls. Despite this intriguing result, the same trend is observed in the morphometric analysis, in which most regions are also considered significant in both cases. Furthermore, the overlap between the significant regions on morphometry versus explainability is high in both cases, specifically, the Jaccard index is 0.74 and 0.85 for WM and the CSF, respectively. Moreover, there is also a high agreement concerning the non-significant regions, as no region was considered significant for either GM or WM modalities when comparing the healthy group with the schizophrenia group and when comparing healthy controls with Alzheimer’s disease patients. In conclusion, the high agreement between the morphometric and sensitivity maps indicate sensitivity analysis maps as a strategy to decode the pathology. Nevertheless, these results should be interpreted with caution, further studies need to be developed to validate this strategy.

BrainAGE encodes disease specificity patterns, and sensitivity maps may disclose structural and morphological differences driven by pathological ageing. Brain age models were trained to tackle the healthy ageing process, and the predicted age of these models yielded statistical differences when comparing healthy controls and clinical groups across three diseases. This finding corroborates the hypothesis that diseases might cause an acceleration of the ageing process.^3^ Sensitivity maps reveal the region’s influence on a prediction, which is different across pathologies and modalities. The comparison of T2D with healthy controls reveals that almost all WM regions exerted different influences on both groups. This result suggests that T2D might be a diffuse pathology in WM, which is consistent with the pathophysiology of T2D, which causes generalized dysfunction of the endothelium and vascular damage.^59^ Regarding Alzheimer’s disease, the sensitivity results evidence that all regions were considered significant regarding the CSF modality, but no region is considered significant on GM, WM and deformation field modalities. These findings suggest that the model explanations on Alzheimer’s disease and T2D were distinct, suggesting that the pathophysiology of both conditions is quite distinct.

Sensitivity maps yield complementary information to morphometry maps. Despite the high agreement between the two approaches, there are also some differences in particular around ventricle regions.

Multiple models retain complementary information to decode the pathology from the age prediction. The minimally processed yields better performance and generalization. Nevertheless, its specificity to detect disease processes is reduced. The results reveal that the pattern is more dissimilar on the other modalities than on minimally processed images. Therefore, the minimally processed model can be used to obtain an accurate measure of the predicted age, yet other modalities might be essential to specify disease mechanisms.

The current study acknowledges some limitations; only SmoothGrad was considered to compute the sensitivity maps. Further studies could explore whether the current results depend on the approach used to explain the model results and the neural network architecture. Furthermore, the explainability of shallow and deep learning across different diseases could be considered. Finally, the current study does not compare the diseases with each other but compares the control group with the clinical condition group. Further investigations are also needed to address this limitation.

## Conclusions

This work highlights the potential of sensitivity maps to uncover the pathological ageing. The results reveal a high agreement between the morphometry and the sensitivity maps, which validates the sensitivity maps as a decoding tool. Furthermore, sensitivity maps yielded distinct patterns across different brain pathologies, highlighting that those predictions encode disease-specific information, and sensitivity maps might be the key to adding specificity to the BrainAGE biomarker. Finally, sensitivity maps can also be used as a complementary strategy to comprehend the biological mechanisms of age-related diseases.

## Supplementary Material

fcaf109_Supplementary_Data

## Data Availability

Eleven open-source data repositories were considered in this work: ABIDE I,^31^ ABIDE II,^32^ GSP,^33^ OASIS-1,^34^ OASIS-2,^35^ OASIS-3,^36^ FCP1000,^37^ ADNI,^38^ CamCAN^39,40^ and the IXI.^41^ The data used for Alzheimer’s disease and T2D that support the findings of this study are available from the corresponding author, upon reasonable request. The code used to conduct this study is available at: https://github.com/mfmachado/brain-age-explainability.
