# Supplementary material for: Unravelling pathological ageing with brain age gap estimation in Alzheimer’s disease, diabetes and schizophrenia
Source: Brain Commun. 2025 Mar 11;7(2):fcaf109. doi: 10.1093/braincomms/fcaf109 (PMC11950532; doi:10.1093/braincomms/fcaf109)
Supplement: fcaf109_Supplementary_Data [file fcaf109_supplementary_data.pdf]

Supplementary material

Maria de Fátima Machado Dias, João Valente Duarte, Paulo Carvalho, Miguel Castelo-Branco

1 Methods

1.1 Data

**Repository**	**Site**	*Total of participants*	Number males	Mean and standard deviation [years]	Min Age [years]	Max Age [years]
ABIDE I	California Institute of Technology	37	30	28.4 ± 10.7	17.0	56.2
ABIDE I	Carnegie Mellon University	26	20	26.8 ± 5.7	19.0	40.0
ABIDE I	Kennedy Krieger Institute	54	41	10.1 ± 1.3	8.1	12.8
ABIDE I	Ludwig Maximilians University Munich	56	49	25.7 ± 11.7	7.0	58.0
ABIDE I	NYU Langone Medical Center	182	145	15.3 ± 6.6	6.5	39.1
ABIDE I	Olin Institute of Livingat Hartford Hospital	16	14	16.9 ± 3.7	10.0	23.0
ABIDE I	Oregon Health and Science University	28	28	10.8 ± 1.9	8.0	15.2
ABIDE I	San Diego State University	36	29	14.4 ± 1.8	8.7	17.2
ABIDE I	Social Brain Lab	28	28	33.4 ± 6.8	20.0	49.0
ABIDE I	Stanford University	37	30	9.9 ± 1.6	7.5	12.9
ABIDE I	Trinity Centre for Health Sciences	49	49	17.2 ± 3.6	12.0	25.9
ABIDE I	University of California Los Angeles	97	86	13.0 ± 2.2	8.4	17.9
ABIDE I	University of Leuven	64	56	18.0 ± 5.0	12.1	32.0
ABIDE I	University of Michigan	143	116	14.0 ± 3.2	8.2	28.8
ABIDE I	University of Pittsburgh School of Medicine	55	48	18.9 ± 6.9	9.3	35.2
ABIDE I	University of Utah School of Medicine	100	100	22.1 ± 7.7	8.8	50.2
ABIDE I	Yale Child Study Center	56	40	12.7 ± 2.9	7.0	17.8
ABIDE II	Barrow Neurological Institute	58	58	38.5 ± 15.5	18.0	64.0
ABIDE II	ETH Zurich	37	37	22.7 ± 4.4	13.8	30.7
ABIDE II	Erasmus University Medical Center Rotterdam	54	44	8.1 ± 1.1	6.2	10.7
ABIDE II	Georgetown University	103	68	10.7 ± 1.6	8.1	13.9
ABIDE II	Indiana University	26	20	24.8 ± 8.5	17.0	54.0
ABIDE II	Institut Pasteur and Robert Debré Hospital	55	25	20.1 ± 10.5	6.1	46.6
ABIDE II	Katholieke Universiteit Leuven	28	28	23.6 ± 4.8	18.0	35.0
ABIDE II	Kennedy Krieger Institute	207	137	10.3 ± 1.3	8.0	13.0
ABIDE II	NYU Langone Medical Center Sample 1	74	67	9.9 ± 5.0	5.2	34.8
ABIDE II	NYU Langone Medical Center Sample 2	27	24	6.8 ± 1.1	5.1	8.8
ABIDE II	Oregon Health and Science University	93	57	10.9 ± 2.0	7.0	15.0
ABIDE II	SanDiego State University	56	47	12.9 ± 3.1	7.4	18.0
ABIDE II	Stanford University	41	37	11.1 ± 1.2	8.4	13.2
ABIDE II	Trinity Centre for Health Sciences	42	42	15.2 ± 3.2	10.0	20.0
ABIDE II	University of California Davis	32	24	14.8 ± 1.8	12.0	17.8
ABIDE II	University of California Los Angeles	31	25	10.8 ± 2.4	7.8	15.0
ABIDE II	University of California Los Angeles Longitudinal Sample	37	35	13.5 ± 1.9	10.0	17.2
ABIDE II	University of Miami	26	20	9.8 ± 2.1	7.1	14.3
ABIDE II	University of Pittsburgh	34	26	14.9 ± 2.4	9.3	19.5
ABIDE II	University of Utah School of Medicine	32	27	20.9 ± 7.9	9.1	38.9
ADNI	–	18705	10233	75.3 ± 7.4	51.0	97.0
GSP	–	1558	661	21.5 ± 2.9	19.0	35.0
OASIS1	–	1683	638	51.5 ± 25.3	18.0	96.0
OASIS2	–	1345	576	76.9 ± 7.6	60.0	98.0
OASIS3	–	2768	1185	70.7 ± 9.3	42.7	97.0
FCP1000	AnnArbor a	25	20	20.4 ± 7.7	13.0	40.0
FCP1000	AnnArbor b	36	17	348.0 ± 1732.7	19.0	9999.0
FCP1000	Atlanta	28	11	30.6 ± 9.2	23.0	54.0
FCP1000	Baltimore	23	8	29.3 ± 5.5	20.0	40.0
FCP1000	Bangor	20	16	22.6 ± 4.6	19.0	38.0
FCP1000	Beijing Zang	197	68	21.1 ± 1.8	18.0	26.0
FCP1000	Berlin Margulies	26	12	29.9 ± 5.2	24.0	44.0
FCP1000	Cambridge Buckner	198	68	20.9 ± 2.1	18.0	29.0
FCP1000	Dallas	24	10	42.9 ± 20.4	20.0	71.0
FCP1000	ICBM	86	0	–	–	–
FCP1000	Leiden 2180	12	9	23.6 ± 2.6	20.0	27.0
FCP1000	Leiden 2200	19	11	21.8 ± 2.7	18.0	28.0
FCP1000	Leipzig	37	13	25.8 ± 5.1	20.0	42.0
FCP1000	Milwaukee a	18	0	–	–	–
FCP1000	Milwaukee b	46	14	53.7 ± 5.9	44.0	65.0
FCP1000	Munchen	16	9	68.3 ± 4.1	63.0	74.0
FCP1000	NYU TRT session1b	12	0	–		
FCP1000	NewHaven a	18	10	31.6 ± 10.3	18.0	48.0
FCP1000	NewHaven b	15	7	27.6 ± 6.4	18.0	42.0
FCP1000	NewYork a	84	40	24.3 ± 10.1	7.0	49.0
FCP1000	NewYork a ADHD	25	18	34.9 ± 9.6	20.0	50.0
FCP1000	NewYork b	20	1	$40.0 \pm nan$	40.0	40.0
FCP1000	Newark	19	9	24.1 ± 3.9	21.0	39.0
FCP1000	Ontario	9	0	–	–	–
FCP1000	Orangeburg	20	12	41.6 ± 11.2	20.0	55.0
FCP1000	Oulu	102	33	21.5 ± 0.6	20.0	23.0
FCP1000	Oxford	22	11	29.3 ± 3.3	21.0	35.0
FCP1000	PaloAlto	17	2	31.6 ± 7.6	22.0	46.0
FCP1000	Pittsburgh	16	9	37.6 ± 8.7	25.0	54.0
FCP1000	Queensland	19	10	25.9 ± 4.1	20.0	34.0
FCP1000	SaintLouis	31	13	25.3 ± 2.3	21.0	29.0
FCP1000	Taipei a	14	0	–	–	–
FCP1000	Taipei b	8	0	–	–	–

Supplementary table 1: Demographics of the participants used to train and validate the autoencoder.

tissue	method	MAE original	MAE corrected	r original	r corrected
min proc	HH	4.46	4.08	**-0.29 ($p = 7.46 \times 10^{-5}$)**	-9.16×10^{-6} ($p = 1.00$)
	IOP	4.66	4.45	**-0.43 ($p = 0.00026$)**	-0.17 ($p = 0.17$)
GM	HH	5.75	4.68	**-0.37 ($p = 2.71 \times 10^{-7}$)**	-3.29×10^{-6} ($p = 1.00$)
	IOP	5.36	4.51	**-0.69 ($p = 8.93 \times 10^{-11}$)**	**-0.43 ($p = 0.00023$)**
WM	HH	5.26	4.01	**-0.46 ($p = 1.37 \times 10^{-10}$)**	-5.20×10^{-6} ($p = 1.00$)
	IOP	6.10	6.48	**-0.78 ($p = 2.60 \times 10^{-15}$)**	**-0.59 (1.51×10^{-7})**
CSF	HH	5.44	4.21	**-0.42 ($p = 4.11 \times 10^{-9}$)**	-5.72×10^{-6} ($p = 1.00$)
	IOP	5.61	5.47	**-0.51 ($p = 8.85 \times 10^{-6}$)**	-0.17 ($p = 0.18$)
DF	HH	6.53	4.14	**-0.57 ($p = 7.45 \times 10^{-17}$)**	1.74×10^{-5} ($p = 1.00$)
	IOP	5.48	6.07	**-0.49 (p=2.56×10^{-5})**	0.044 ($p = 0.72$)

Supplementary table 2: MAE and the pearson correlation between BrainAGE and age, with and without the bias correction.

2 Results

2.1 MAE and BAG

2.1.1 Schizophrenia

Metric [in years]	Clinical condition	Minimally processed	GM	WM	CSF	Deformation fields
MAE	Control	3.56	4.10	4.09	4.45	4.16
	Schizophrenia	**4.79**	**5.30**	**6.09**	**5.64**	**6.22**
BAG	Control	-0.01	1.12	2.09	1.98	1.13
	Schizophrenia	2.39	2.59	4.91	3.90	3.50

Supplementary table 3: MAE and mean of BrainAGE, in years, results of the brain age models for the schizophrenia analysis.

2.1.2 Diabetes

Metric [in years]	Clinical group	Minimally processed	GM	WM	CSF	Deformation fields
MAE	Control	4.44	4.24	4.71	4.79	4.56
	T2D	**6.43**	**8.59**	**7.86**	**7.71**	**11.13**
BAG mean	Control	-3.17	0.57	0.88	-1.21	0.90
	T2D	3.58	8.24	6.47	5.99	9.37

Supplementary table 4: MAE and mean of BrainAGE, in years, results of the brain age models for the T2D analysis.

2.1.3 Alzheimer

Metric [in years]	Clinical condition	Minimally processed	GM	WM	CSF	Deformation fields
MAE	Control	5.44	5.87	4.12	5.85	6.95
	AD	**6.36**	**11.43**	**8.41**	**10.31**	**12.70**
BAG	Control	-3.81	1.25	0.98	-0.68	3.37
	AD	5.23	11.21	8.41	9.01	12.41

Supplementary table 5: MAE and mean of BrainAGE, in years, results of the brain age models for the AD dataset.

2.2 Morphometry and sensitivity maps analysis

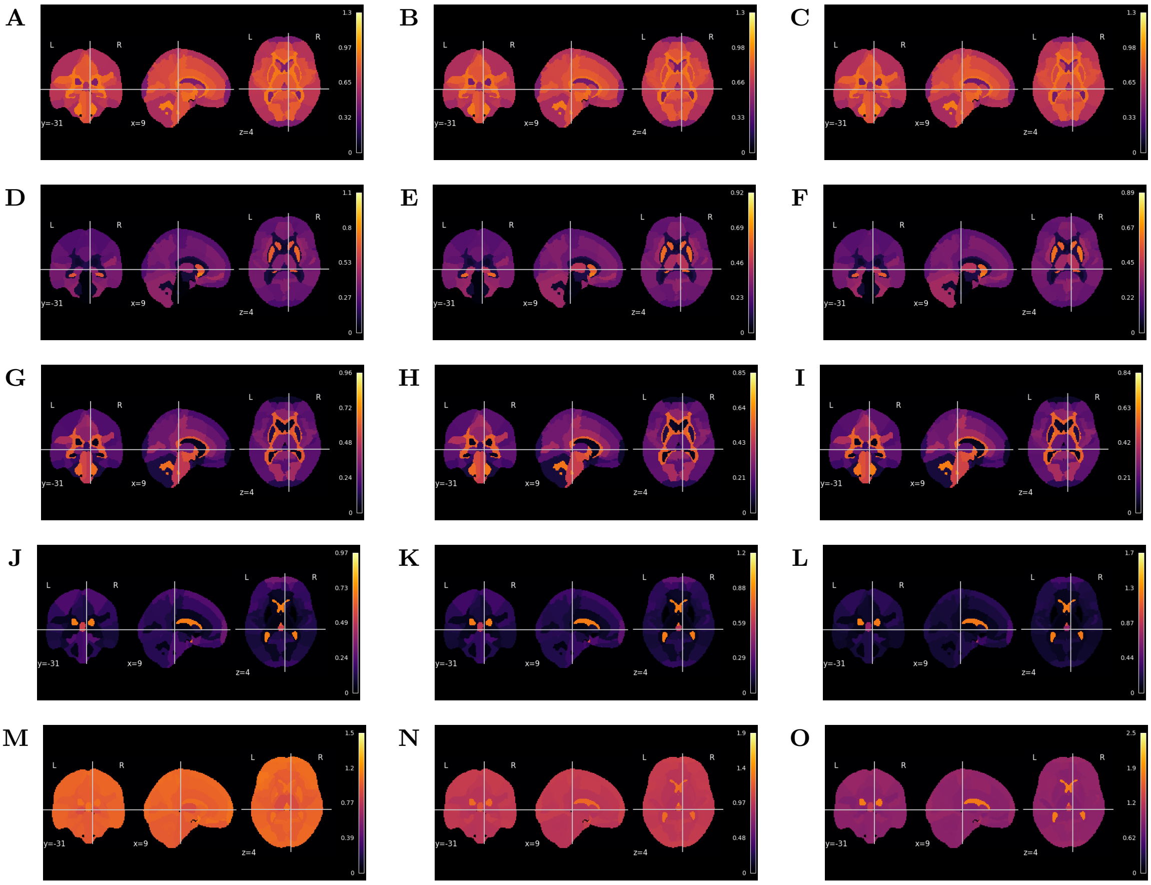

Supplementary figure 1: ***Mean of the morphometry map values per ROI.*** *Mean of the morphometry map values regardless of the pathology. The A, D, G, J, and M corresponds to the to the mean of the schizophrenia dataset. B, E, H, K, and N to the Type 2 Diabetes (T2D) datset. And C, F, I, L and O to the Alzheimer's Disease (AD). Different morphometric mean maps were derived from the minimally processed (A, B, and C), grey matter (D, E, and F), white matter (G, H, and I), Cerebrospinal fluid (J, K, and L) and deformation fields (M, N, and O).*

	Schizophrenia	T2D	AD
Minimally processed	73.57	57.86	8.57
GM	94.29	84.29	0.00
WM	4.29	63.57	0.00
CSF	95.00	91.43	0.00
DF	66.43	41.43	0.00

Supplementary table 6: Percentage ROIs which were considered significant for the age factor on the ANCOVA test on the morphometrics analysis.

	Schizophrenia	T2D	AD
Minimally processed	100.00	100.00	99.29
GM	100.00	72.14	0.00
WM	97.86	98.57	0.00
CSF	100.00	10.00	0.00
DF	54.29	60.71	30.00

Supplementary table 7: Percentage of the ROIs which were considered significant for the age factor on the ANCOVA test on the sensitivity map analysis.

	Schizophrenia	T2D	AD
Minimally processed	0.74	0.58	0.09
GM	0.94	0.72	0.00
WM	0.04	0.62	0.00
CSF	0.95	0.07	0.00
DF	0.41	0.31	0.00

Supplementary table 8: Jaccard index comparing the significant ROIs on age factor of morphometric with the sensitivity analysis.

	Schizophrenia	T2D	AD
Minimally processed	20.00	34.29	30.00
GM	1.43	61.43	0.00
WM	0.00	74.29	0.00
CSF	56.43	33.57	85.00
DF	12.14	11.43	30.00

Supplementary table 9: Percentage ROIs which were considered significant for the clinical condition factor on the ANCOVA test on the morphometrics analysis.

	Schizophrenia	T2D	AD
Minimally processed	80.00	97.86	69.29
GM	0.00	18.57	0.00
WM	0.00	94.29	0.00
CSF	0.00	0.00	100.00
DF	0.00	26.43	0.00

Supplementary table 10: Percentage of ROIs which were considered significant for the clinical condition factor on the ANCOVA test on the sensitivity map analysis.

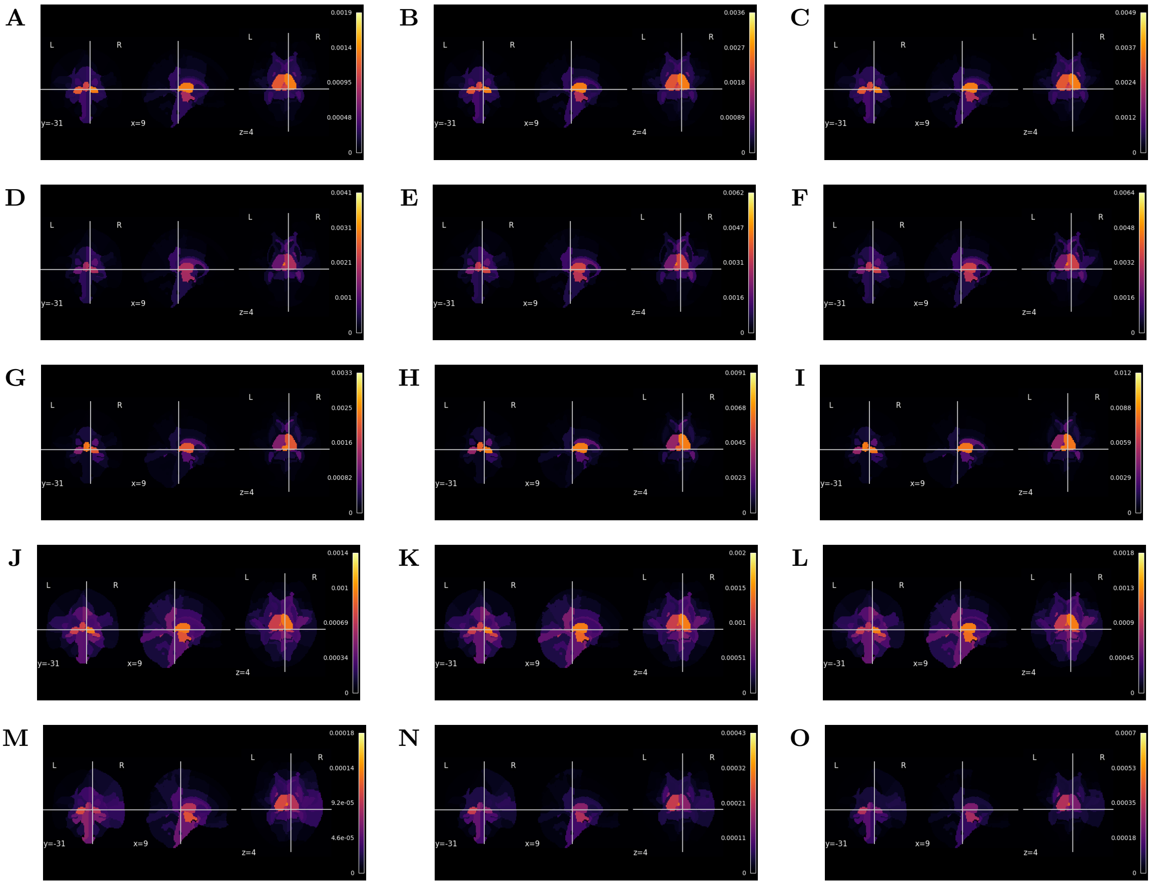

Supplementary figure 2: ***Mean of the explainability map values per ROI.*** *Mean of the explainability map values regardless of the pathology. The A, D, G, J, and M corresponds to the to the mean of the schizophrenia dataset. B, E, H, K, and N to the Type 2 Diabetes (T2D) datset. And C, F, I, L and O to the Alzheimer's Disease (AD). Different explainability mean maps were derived from the minimally processed (A, B, and C), grey matter (D, E, and F), white matter (G, H, and I), Cerebrospinal fluid (J, K, and L) and deformation fields (M, N, and O).*

	Schizophrenia	T2D	AD
Minimally processed	0.74	0.58	0.09
GM	0.94	0.72	0.00
WM	0.04	0.62	0.00
CSF	0.95	0.07	0.00
DF	0.41	0.31	0.00

Supplementary table 11: Jaccard index comparing the significant ROIs on age factor of morphometric with the sensitivity analysis.

2.3 OASIS4

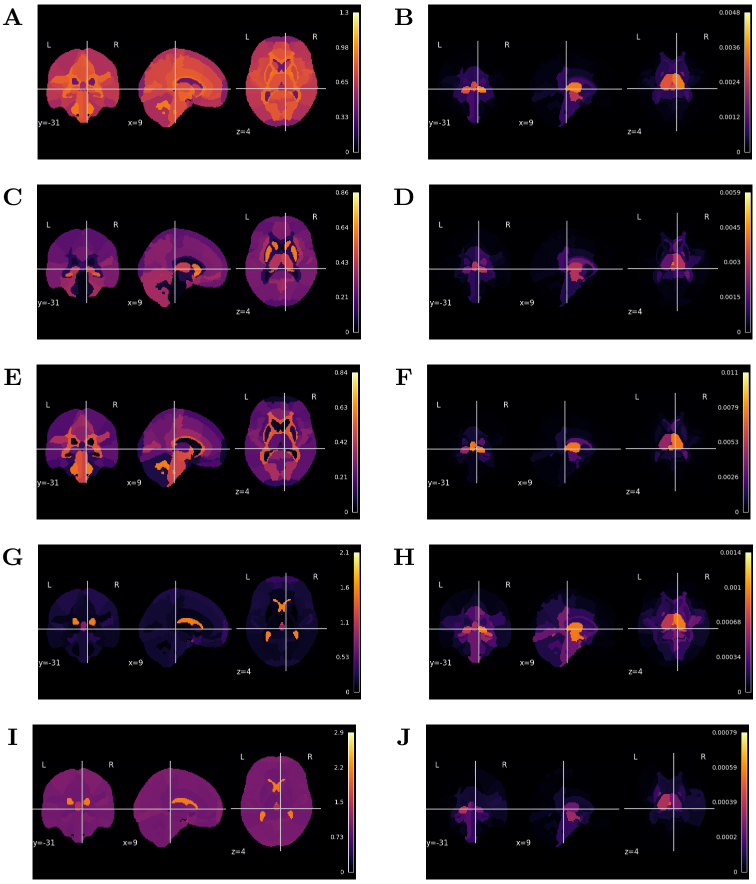

Supplementary figure 3: ***Mean of the image type per ROI for the OASIS4 dataset.*** *Mean of the morphometrics (A, C, E, G, I) explainability map (B, D, F, H, J) values regardless of the pathology for the OASIS4 dataset. The image types are minimally processed image (A, B), grey matter (C, D), white matter (E, F), cerebrospinal fluid (G, H) e deformation fields (I, J).*

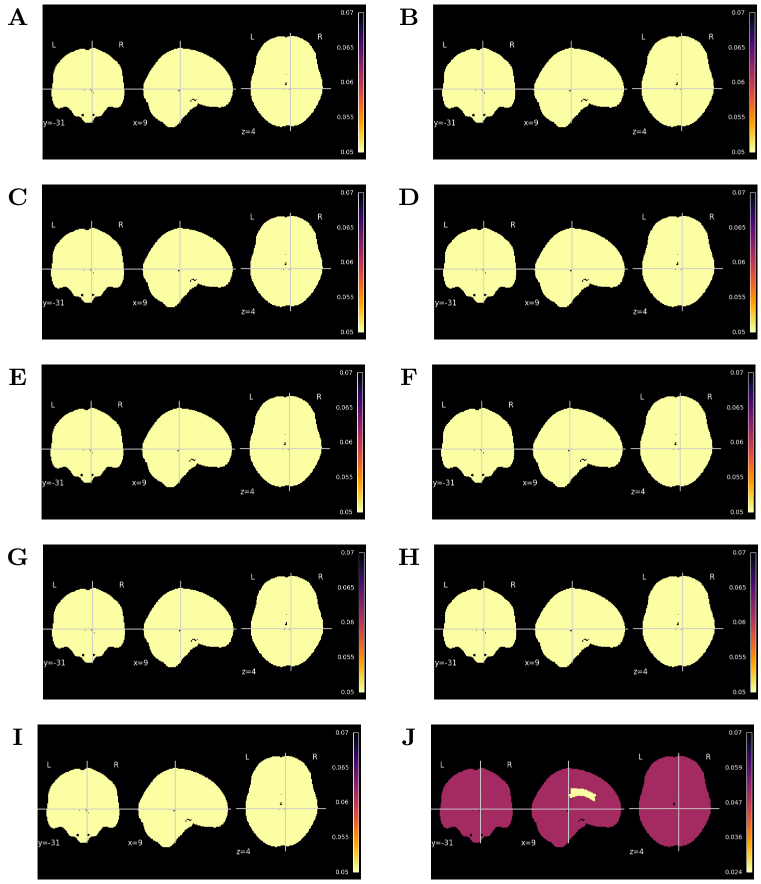

Supplementary figure 4: Region-of-interest (ROI) ANCOVA p-value results for the age factor. The ANCOVA compared the morphometric map ROI mean of clinical conditions [46 health controls/ 46 pathology] and controlling for age. ANCOVA results for morphometric maps (A, C, E, G, I) explainability maps (B, D, F, H, J). The image types are minimally processed image (A, B), grey matter (C, D), white matter (E, F), cerebrospinal fluid (G, H) e deformation fields (I, J).

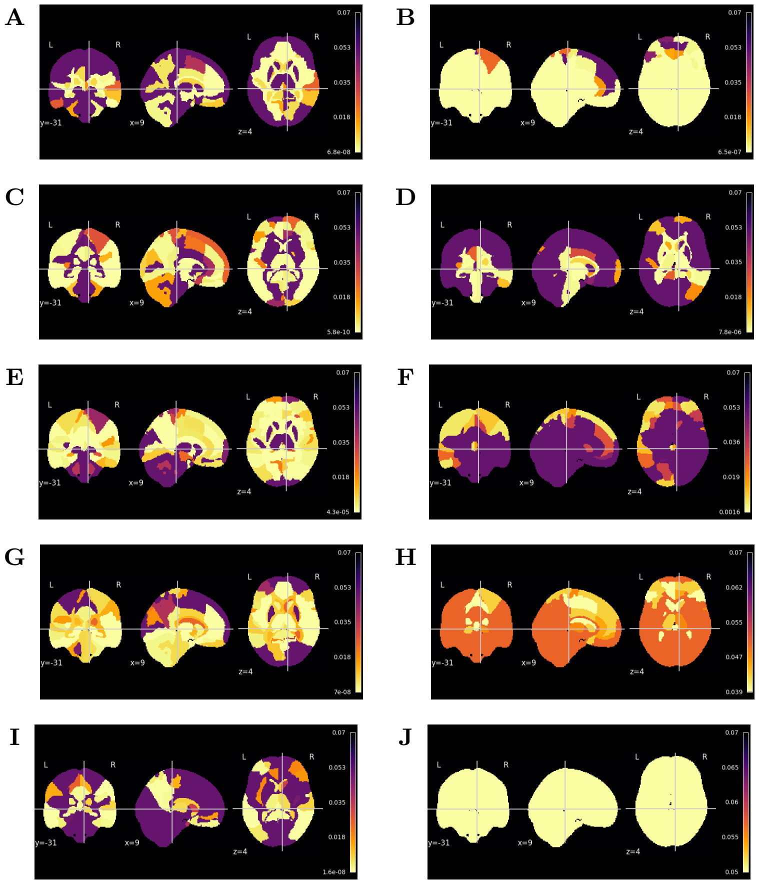

Supplementary figure 5: Region-of-interest (ROI) ANCOVA p-value results for the clinical condition factor. The ANCOVA compared the morphometric map ROI mean of clinical conditions [46 health controls/ 46 pathology] and controlling for age. ANCOVA results for morphometric maps (A, C, E, G, I) explainability maps (B, D, F, H, J). The image types are minimally processed image (A, B), grey matter (C, D), white matter (E, F), cerebrospinal fluid (G, H) e deformation fields (I, J).

	Local AD dataset	OASIS4
Minimally processed	8.57	0.00
GM	0.00	0.00
WM	0.00	0.00
CSF	0.00	0.00
DF	0.00	0.00

Supplementary table 12: Percentage ROIs which were considered significant for the age factor on the ANCOVA test on the morphometrics analysis. Results for the local Alzheimer' Disease (AD) and OASIS4 datasets.

	Local AD dataset	OASIS4
Minimally processed	99.29	0.00
GM	0.00	0.00
WM	0.00	0.00
CSF	0.00	0.00
DF	30.00	71.43

Supplementary table 13: Percentage of the ROIs which were considered significant for the age factor on the ANCOVA test on the sensitivity map analysis. Results for the local Alzheimer' Disease (AD) and OASIS4 datasets.

	Local AD dataset	OASIS4
Minimally processed	30.00	45.00
GM	0.00	72.86
WM	0.00	74.29
CSF	85.00	80.00
DF	30.00	46.43

Supplementary table 14: Percentage ROIs which were considered significant for the clinical condition factor on the ANCOVA test on the morphometrics analysis. Results for the local Alzheimer' Disease (AD) and OASIS4 datasets.

	Local AD dataset	OASIS4
Minimally processed	69.29	95.00
GM	0.00	24.26
WM	0.00	33.57
CSF	100.00	31.43
DF	0.00	0.00

Supplementary table 15: Percentage of ROIs which were considered significant for the clinical condition factor on the ANCOVA test on the sensitivity map analysis. Results for the local Alzheimer' Disease (AD) and OASIS4 datasets.

2.4 Noise impact on sensitivity maps per tissue

The relation of correlation between sensitivity and age with noise is depicted in Figure 6 for the different modalities. The results reveal that each tissue achieves the maximum correlation at a different noise level. Moreover, the correlation evolution is specific to each tissue. WM peaks at 2% and decreases drastically afterwards, the GM and deformation fields exhibit a smoother relationship with noise, reaching their maximum at a noise level of 2% and 4%, respectively. Minimally processed images plateau between 10% and 20%, reaching their maximum at 28% and decreasing more abruptly subsequently. CSF experiences an abrupt transition to the maximum at 6%, with a smoother decrease afterwards. The maximum correlation values attained are 0.87 for minimally processed images, followed by GM, WM, and CSF with 0.82, 0.80, 0.63, and deformation fields with 0.30, respectively.

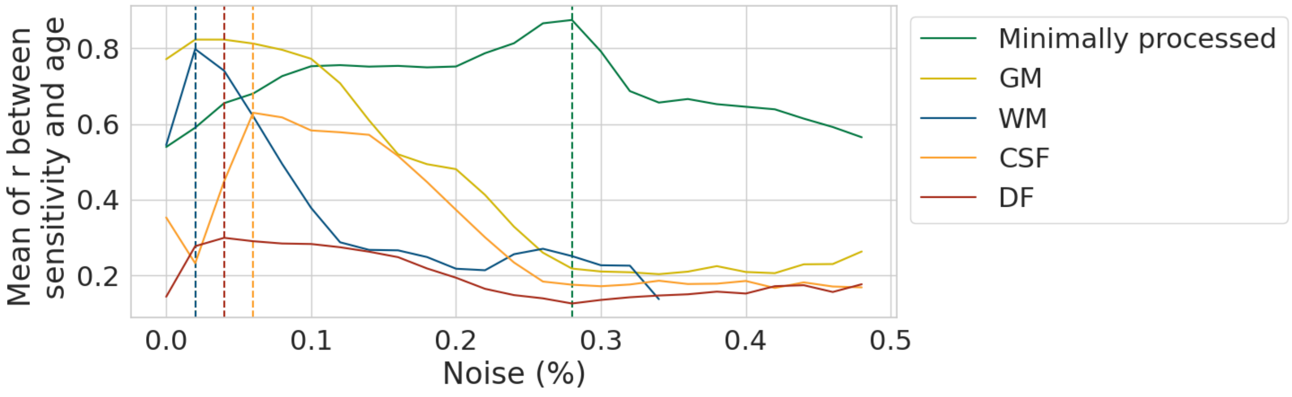

Supplementary figure 6: Evolution of the Pearson correlation means between saliency and age for different noise levels using the IXI–HH data.

The correlation between sensitivity and age varies, drastically, with different noise degrees. Furthermore, the correlation of evolution with age depends upon the modality. This finding is in agreement with the results reported by Smilkov *et al*[1], the most suitable noise level depends upon the input type. For natural images, it is advantageous to apply noise within the 10-25% range, while the MNIST dataset (black and white images) exhibited optimal results at approximately 50%. Similarly, in these cases, GM, WM, CSF and deformation field images require reduced noise values, while minimally processed images require higher levels of noise. Moreover, in our results, minimally processed seems to be the modality more robust to noise.

Sensitivity maps yield reproducible results across datasets concerning the regions correlated with age. A perfect agreement is reported between the baseline (IXI-HH set) and the Cobre dataset. Regarding the diamarker, the agreement is perfect on minimally processed images and WM and very high on GM and deformation fields. Finally, for the AD dataset, the agreement with baseline results is also perfect for the minimally processed image, but not for the other modalities. The morphometric analysis also yields reproducible results between the baseline dataset and Diamarker and Cobre. The AD dataset yields poor agreement with the baseline regarding the significant regions. As previously discussed this result might be related to the age range and the reduced number of samples of the AD dataset.

2.5 High resolution images

[1]Smilkov, Daniel: SmoothGrad: removing noise by adding noise, in: (2017).

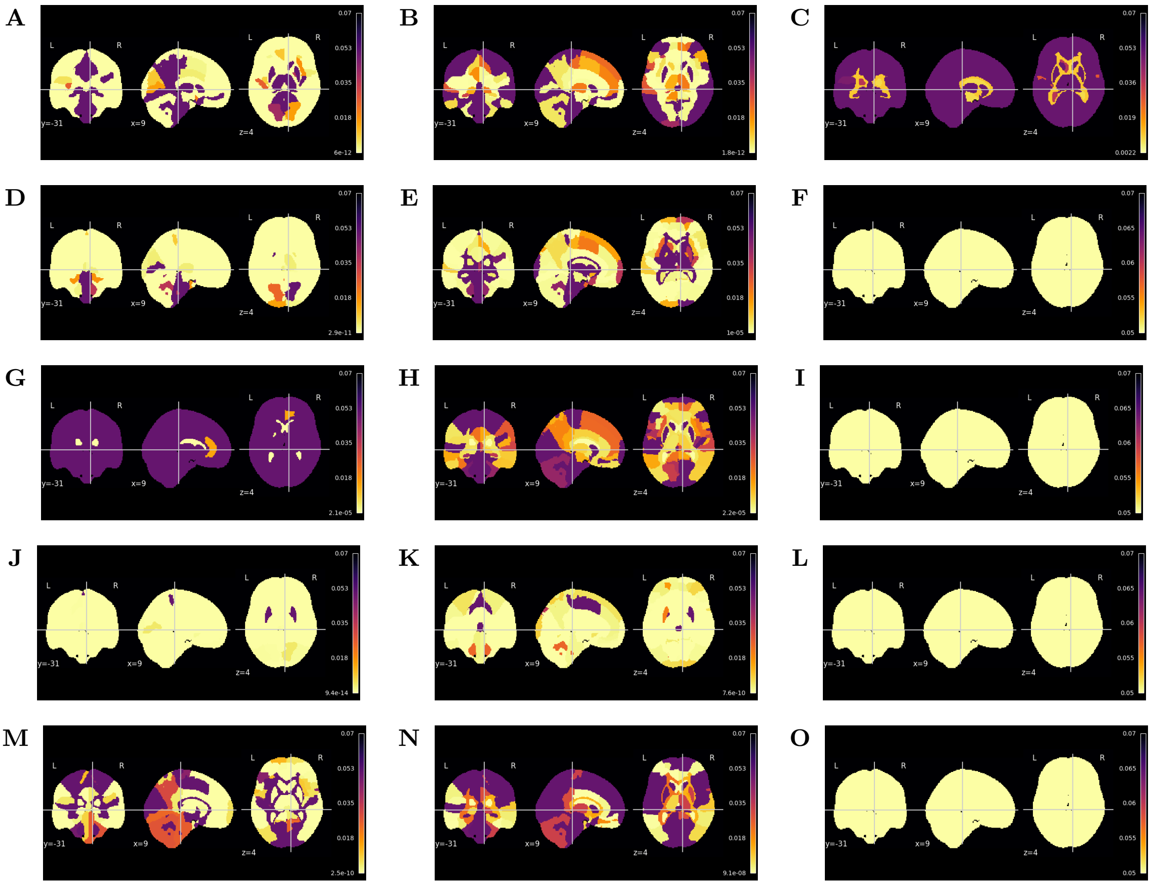

Supplementary figure 7: Region-of-interest (ROI) ANCOVA p-value results for age on morphometric maps. The ANCOVA compared the morphometric map ROI mean of clinical conditions [health controls/pathology] and controlling for age. The pathologies assessed were Schizophrenia (A, D, G, J, and M) [72 controls /72 schizophrenia], Type 2 Diabetes (T2D) (B, E, H, K, and N) [82 controls /70 T2D], and Alzheimer's Disease (AD) (C, F, I, L and O) [18 controls /20 AD]. Different morphometric maps assessed were minimally processed (A, B, and C), grey matter (D, E, and F), white matter (G, H, and I), Cerebrospinal fluid (J, K, and L), and deformation fields (M, N, and O).

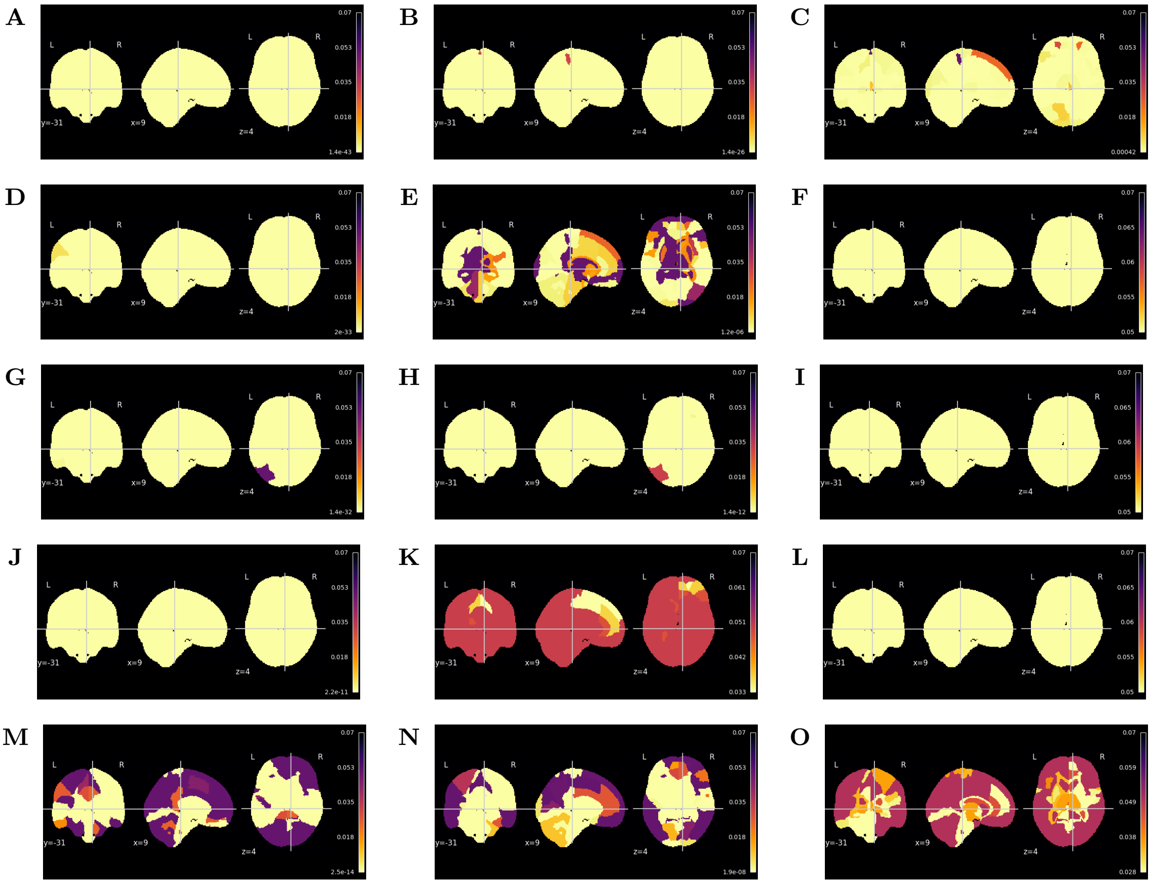

Supplementary figure 8: Region-of-interest (ROI) ANCOVA p-value results for age on sensitivity maps. The ANCOVA compared the morphometric map ROI mean of clinical conditions (health controls versus pathology) and controlling for age. The pathologies assessed were Schizophrenia (A, D, G, J, and M) [72 controls /72 schizophrenia], Type 2 Diabetes (T2D) (B, E, H, K, and N) [82 controls /70 T2D], and Alzheimer's Disease (AD) (C, F, I, L and O) [18 controls /20 AD]. Different sensitivity maps derived from brain-age models trained with minimally processed (A, B, and C), grey matter (D, E, and F), white matter (G, H, and I), Cerebrospinal fluid (J, K, and L) and deformation fields (M, N, and O) were assessed.

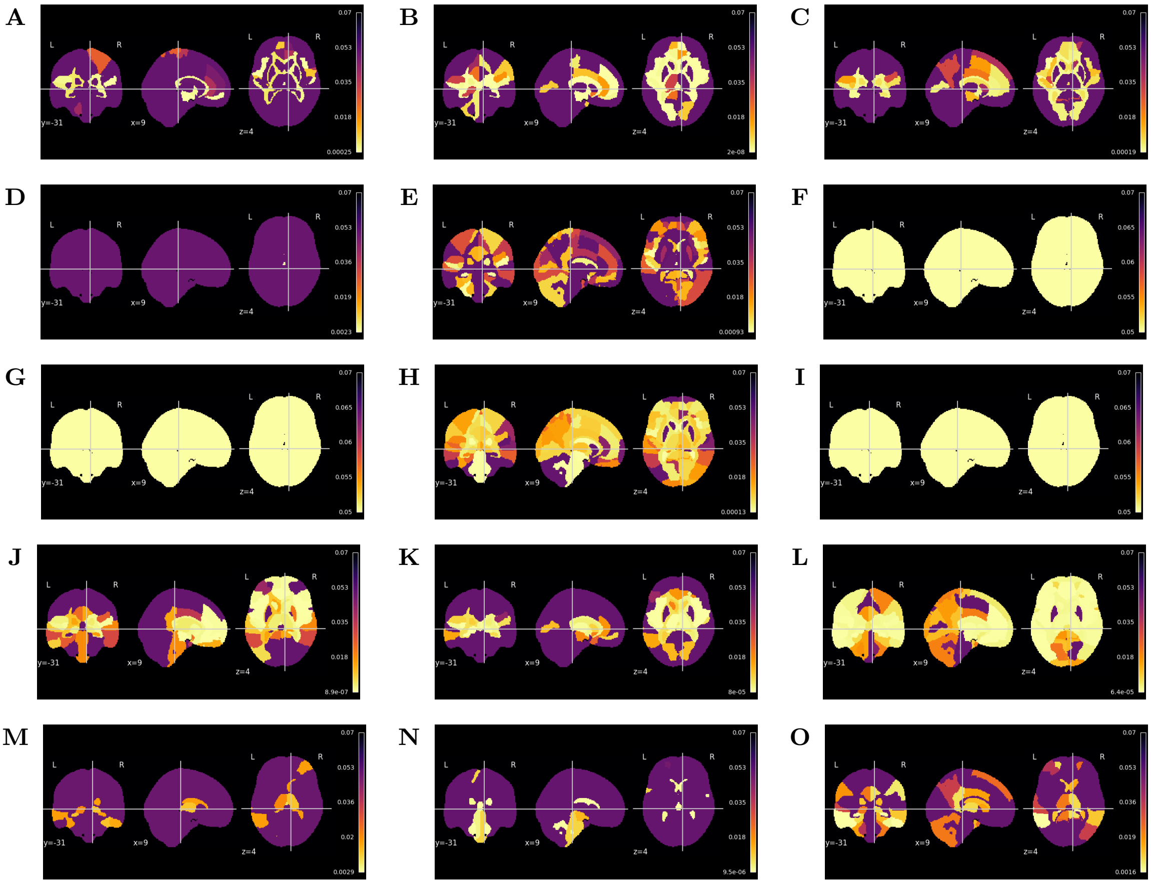

Supplementary figure 9: Region-of-interest (ROI) ANCOVA p-value results for clinical condition on morphometric maps. The ANCOVA compared the morphometric map ROI mean of clinical conditions (health controls versus pathology) and controlling for age. The pathologies assessed were Schizophrenia (A, D, G, J, and M) [72 controls /72 schizophrenia], Type 2 Diabetes (T2D) (B, E, H, K, and N) [82 controls /70 T2D], and Alzheimer's Disease (AD) (C, F, I, L and O) [18 controls /20 AD]. Different morphometric maps assessed were minimally processed (A, B, and C), grey matter (D, E, and F), white matter (G, H, and I), Cerebrospinal fluid (J, K, and L) and deformation fields (M, N, and O).

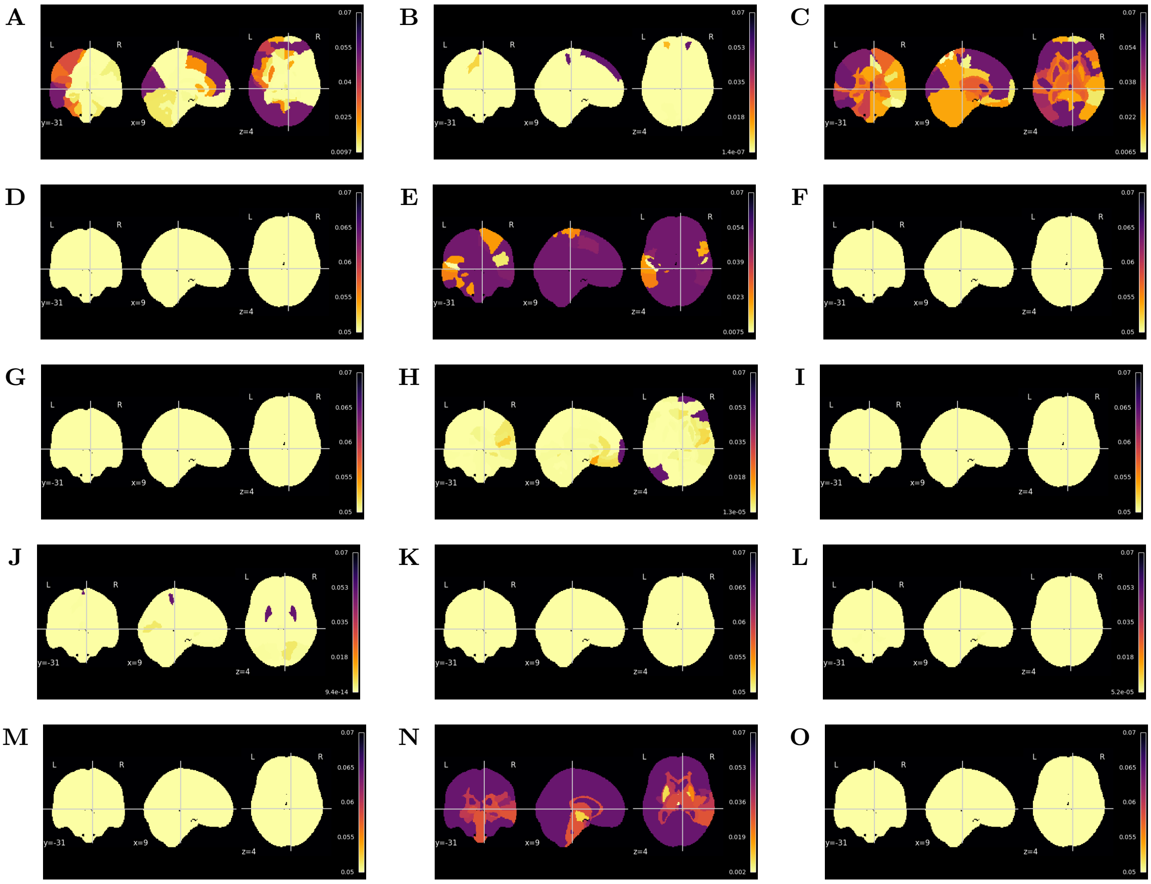

Supplementary figure 10: Region-of-interest (ROI) ANCOVA p-value results for clinical condition on sensitivity maps. The ANCOVA compared the sensitivity map ROI mean of clinical conditions (health controls versus pathology) and controlling for age. The pathologies assessed were Schizophrenia (A, D, G, J, and M) [72 controls /72 schizophrenia], Type 2 Diabetes (T2D) (B, E, H, K, and N) [82 controls /70 T2D], and Alzheimer's Disease (AD) (C, F, I, L and O) [18 controls /20 AD]. Different sensitivity maps derived from brain-age models trained with minimally processed (A, B, and C), grey matter (D, E, and F), white matter (G, H, and I), Cerebrospinal fluid (J, K, and L) and deformation fields (M, N, and O) were assessed.

References

SMILKOV, Daniel: SmoothGrad: removing noise by adding noise, in: (2017) URL: https://arxiv.org/abs/1706.03825v1.
